# Mutation in the Plasmodium falciparum BTB/POZ Domain of K13 Protein Confers Artemisinin Resistance

**DOI:** 10.1128/AAC.01320-21

**Published:** 2022-01-18

**Authors:** Lucie Paloque, Romain Coppée, Barbara H. Stokes, Nina F. Gnädig, Karamoko Niaré, Jean-Michel Augereau, David A. Fidock, Jérôme Clain, Françoise Benoit-Vical

**Affiliations:** a LCC-CNRS, Laboratoire de Chimie de Coordination, Université de Toulouse, CNRS, Toulouse, France; b MAAP, New Antimalarial Molecules and Pharmacological Approaches, Toulouse, France; c Institut de Pharmacologie et de Biologie Structurale, IPBS, Université de Toulouse, CNRS, UPS, Toulouse, France; d Université de Paris, MERIT, IRD, Paris, France; e Department of Microbiology and Immunology, Department of Medicine, Columbia University grid.239585.0grid.21729.3f Irving Medical Center, New York, New York, USA; f Malaria Research and Training Center, University of Sciences, Techniques and Technologies of Bamako, Bamako, Mali; g Division of Infectious Diseases, Department of Medicine, Columbia University grid.239585.0grid.21729.3f Irving Medical Center, New York, New York, USA; h Centre National de Référence du Paludisme, Laboratoire de Parasitologie, Hôpital Bichat-Claude Bernard, HUPNVS, APHP, Paris, France

**Keywords:** malaria, artemisinin resistance, K13 mutation, BTB/POZ domain

## Abstract

Partial artemisinin resistance, defined in patients as a delayed parasite clearance following artemisinin-based treatment, is conferred by non-synonymous mutations in the Kelch beta-propeller domain of the Plasmodium falciparum *k13* (*pfk13*) gene. Here, we carried out *in vitro* selection over a 1-year period on a West African P. falciparum strain isolated from Kolle (Mali) under a dose-escalating artemisinin regimen. After 18 cycles of sequential drug pressure, the selected parasites exhibited enhanced survival to dihydroartemisinin in the ring-stage survival assay (RSA^0-3h^ = 9.2%). Sanger and whole-genome sequence analyses identified the PfK13 P413A mutation, localized in the BTB/POZ domain, upstream of the propeller domain. This mutation was sufficient to confer *in vitro* artemisinin resistance when introduced into the PfK13 coding sequence of the parasite strain Dd2 by CRISPR/Cas9 gene editing. These results together with structural studies of the protein demonstrate that the propeller domain is not the sole *in vitro* mediator of PfK13-mediated artemisinin resistance, and highlight the importance of monitoring for mutations throughout PfK13.

## INTRODUCTION

Artemisinins (ART, meaning artemisinin and its derivatives) are the core components of the current front-line artemisinin-based combination therapies (ACTs) that are used to treat Plasmodium falciparum malaria (https://www.who.int/publications/i/item/9789240015791, WHO 2020). However, P. falciparum parasites with reduced sensitivity to ART, i.e., partial resistance, have emerged and spread throughout the Greater Mekong Subregion during the last decade (1–3). Clinical ART resistance has also been reported in India (4, 5) and Papua New Guinea (6), and is highly suspected in South America (7) and East Africa (8–11). It has been estimated that by 2040, African malaria-endemic countries (which account for 94% of malaria cases and deaths, most of which occur in children aged under 5 years) might face widespread ART resistance. Such a scenario would be a major public health disaster (12) (https://www.who.int/publications/i/item/9789240015791, WHO 2020). ART resistance is defined by a delayed parasite clearance half-life (PCt_½_ > 5 h) *in vivo* following an ART-based treatment (2), or by an increased parasite survival rate following a brief exposure to a high dose of dihydroartemisinin (DHA) in the ring-stage survival assay (RSA^0-3h^) *in vitro* (13). The first ART-resistant laboratory strain (F32-ART5) was selected through *in vitro* exposure to a dose-escalating sequential ART regimen (14) and led to the identification of the M476I mutation in the parasite’s *pfk13* gene as the main driver of resistance (15). This gene encodes a 726-amino acid protein (PfK13) containing three highly conserved domains: a coiled-coil-containing domain, a BTB/POZ domain, and a Kelch-repeat beta-propeller domain (16–18). *In vitro* selection of ART resistance may also produce a K13-independent resistance phenotype as previously shown (19, 20). However, in clinical isolates, only non-synonymous single nucleotide polymorphisms (SNPs) in the *pfk13* gene have been correlated with ART resistance, almost all of which have been located in the propeller domain (15). Since this discovery, clinical surveys and epidemiological studies have investigated genetic variation in *pfk13* across many malaria-endemic areas, mostly focusing on mutations in the propeller domain (3, 6, 10, 21–24). Based on the mutation frequency and the *in vitro* or *in vivo* phenotype, the World Health Organization lists some 20 validated or candidate mutations in the *pfk13* propeller domain as molecular markers of ART resistance (https://www.who.int/publications/i/item/9789240012813, WHO 2020).

Here, we carried out an *in vitro* resistance selection with increasing concentrations of ART over a 1-year period on a previously isolated West African P. falciparum strain from Kolle, Mali (25, 26). Our *in vitro* selection yielded a parasite lineage exhibiting enhanced survival to DHA as measured by RSA^0-3h^. This resistance phenotype was attributed to the *pfk13* mutation P413A and was confirmed by CRISPR/Cas9 gene editing in Dd2 parasites. This mutation is not located in the propeller domain but in the BTB/POZ domain of PfK13, highlighting the importance of monitoring mutations in other PfK13 domains.

## RESULTS

### Selection of ART resistance and phenotypic characterization of a novel ART-resistant strain.

**FIG 1:**
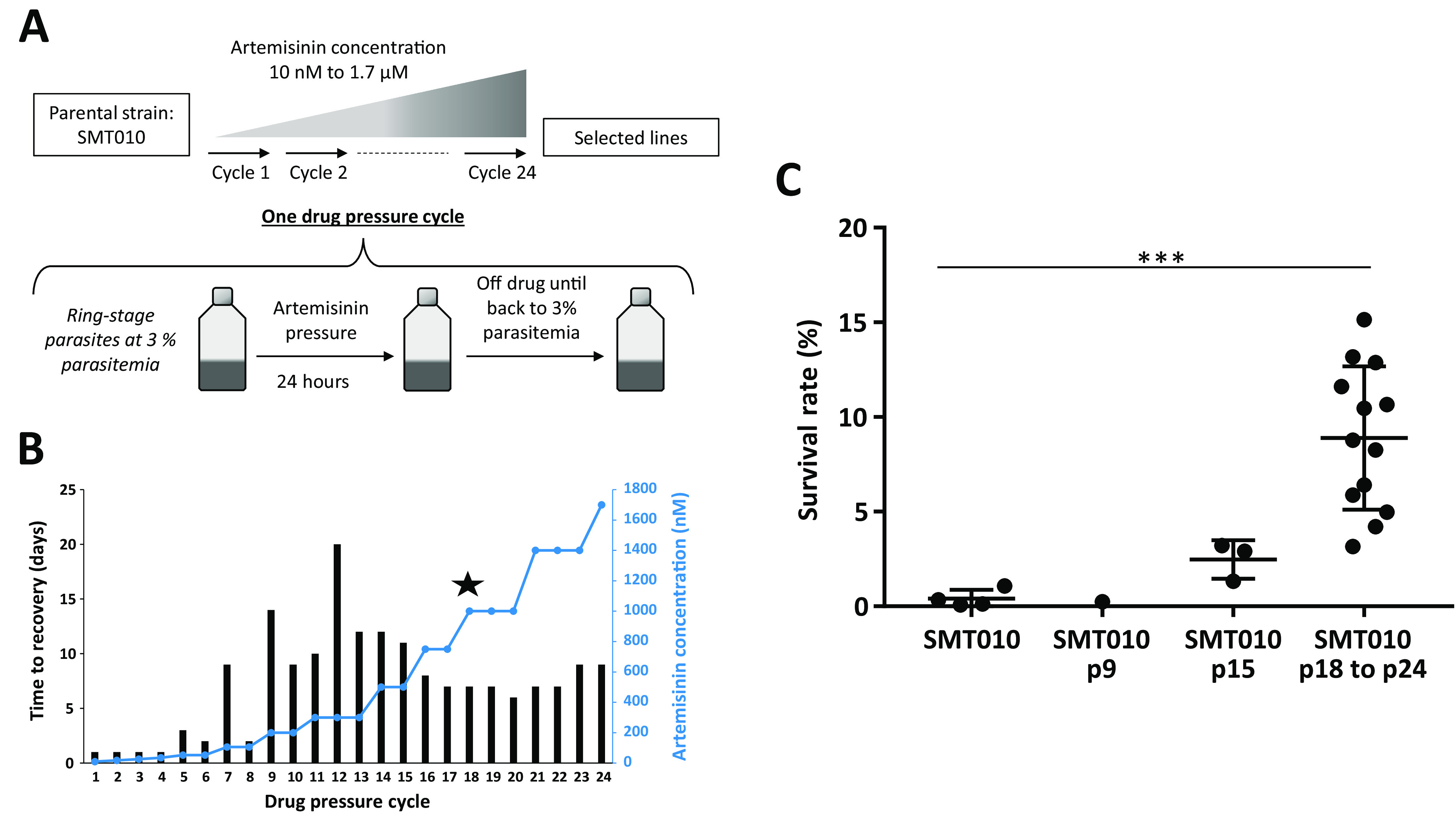
(A) *In vitro* selection of ART resistance by exposure of the parental field isolate SMT010 to escalating drug pressure cycles. Each cycle of drug pressure exposed ring-stage parasites at 3% parasitemia to sequential and increasing doses of ART for 24 h. After drug removal, parasites were placed back in drug-free culture conditions until they reached 3% parasitemia. Selected lineages are annotated SMT010pX (“p” for pressure and “X” for the number of pressure cycles). (B) ART concentration applied for each pressure cycle across the year of selection process (blue line) and the corresponding time to recovery of parasites (time necessary to go back to 3% parasitemia) after the drug pressure (black bars). The star indicates the appearance at p18 of the P413A mutation in the *pfk13* gene. (C) RSA^0-3h^ survival rates 72 h after exposure to a 6 h pulse of 700 nM dihydroartemisinin (DHA) for the parental strain SMT010 and the selected lineages (all the selected lineages after 18 pressure cycles of drug pressure are K13-mutated). Mean survival rates and numbers of independent experiments for each strain are presented in supplemental Table S1. *P* values were derived by using the Mann-Whitney *U* test, comparing selected strains to the parental strain. *** *P* < 0.001.

The field parasite isolate SMT010 (randomly chosen) used in this study was collected in Mali in 2010. This isolate was not associated with any clinical failure after an ACT treatment course, and displayed a wild-type *pfk13* sequence and an *in vitro* RSA^0-3h^ survival rate < 1% (25). Whole-genome sequence analysis of SMT010 indicated that this strain is monoclonal (Table S1). The strain SMT010 was exposed to intermittent and increasing ART concentrations (from 10 nM to 1.7 μM). For each cycle of ART pressure, ring-stage parasites at 3% parasitemia were exposed to ART for 24 h and then cultured in drug-free medium until reaching 3% parasitemia, a process that took 20 days at most, for each concentration (Fig. 1A and B). Twenty-four drug pressure cycles were performed, and the selection process lasted 1 year. Across this selection process, the DHA sensitivity of the derived lineages was assessed by RSA^0-3h^ on a regular basis. After 15 cycles of ART pressure (ART concentration at cycle 15: 500 nM), the line SMT010p15 (“p” for “pressure”) displayed an increased RSA^0-3h^ survival rate of 2.5%, but this value was not significantly different compared to the parental strain (*P* value = 0.057, Mann-Whitney *U* test; Fig. 1C and Table S2). After 18 cycles of ART pressure (ART concentration at cycle 18: 1 μM), a parasite line with significantly reduced ART susceptibility, SMT010p18, was isolated. This line exhibited an RSA^0-3h^ survival rate of 9.2%, which then varied from 6.9 to 11.6% across the next six cycles of drug pressure. The group of lines selected from p18 to p24 exhibited RSA^0-3h^ survival rates that were significantly higher compared to the parental strain (*P* value = 8.0 × 10^−4^, Mann-Whitney *U* test; Fig. 1C and Table S2). Interestingly, the RSA values of the selected lines were similar to those previously observed for culture-adapted isolates harboring *pfk13* ART resistance mutations (15, 18). As has been previously observed for ART resistance (14), there were no significant changes in ART IC_50_ values between the parental and selected lineages, as assessed by a standard growth inhibition assay (*P* value = 0.107; Table S3).

### Genotypic characterization of the selected line.

Sanger sequencing of the *pfk13* gene identified the P413A (codon CCG → GCG) mutation in SMT010p18 and SMT010p24 lines, but not in SMT010p15. To confirm this, we compared the whole-genome sequence of SMT10p19 with the parental strain SMT010 (Fig. S1, File S1). SNPs located in the variable subtelomeric regions (including *var*, *stevor*, *rifin*, *phist,* and *Plasmodium* exported protein-encoding genes) were discarded, considering the high rate of mapping error. A total of 11 non-synonymous SNPs distributed across seven genes were identified (Table 1). A single SNP was found in the five genes coding for each of the following proteins: PP-loop family protein (PF3D7_0411200), rhoptry protein RHOP148 (PF3D7_1366400), PfK13 protein (PF3D7_1343700), and two conserved proteins of unknown function. Two SNPs were found in the gene coding for a zinc finger protein (PF3D7_1425600) and four SNPs in the gene coding for the erythrocyte vesicle protein 1 (PF3D7_0410000). No copy number differences were observed between SMT010 and SMT010p19 (Fig. S2). We decided to focus our efforts on the P413A mutation in *pfk13*.

**TABLE 1:** Candidate genes identified by whole-genome sequencing in the selected lineage SMT010p19 (the complete list of the genetic variations existing between SMT010 and SMT010p19 is available in Fig. S1 and File S1 in the supplemental material)

Gene ID	Gene name	SNPs in SMT010p19
PF3D7_0410000	Erythrocyte vesicle protein 1	K294E, E286V, T290R, N296H
PF3D7_0411200	PP-loop family protein	K1056I
PF3D7_0710200	Conserved protein unknown function	N693S
**PF3D7_1343700**	**Kelch protein K13**	**P413A**
PF3D7_1362700	Conserved protein unknown function	D1080N
PF3D7_1366400	Rhoptry protein RHOP148	T285I
PF3D7_1425600	Zinc finger protein	H1497N, N1505H

### Validation of the role of P413A mutation in conferring *in vitro* ART resistance.

**FIG 2:**
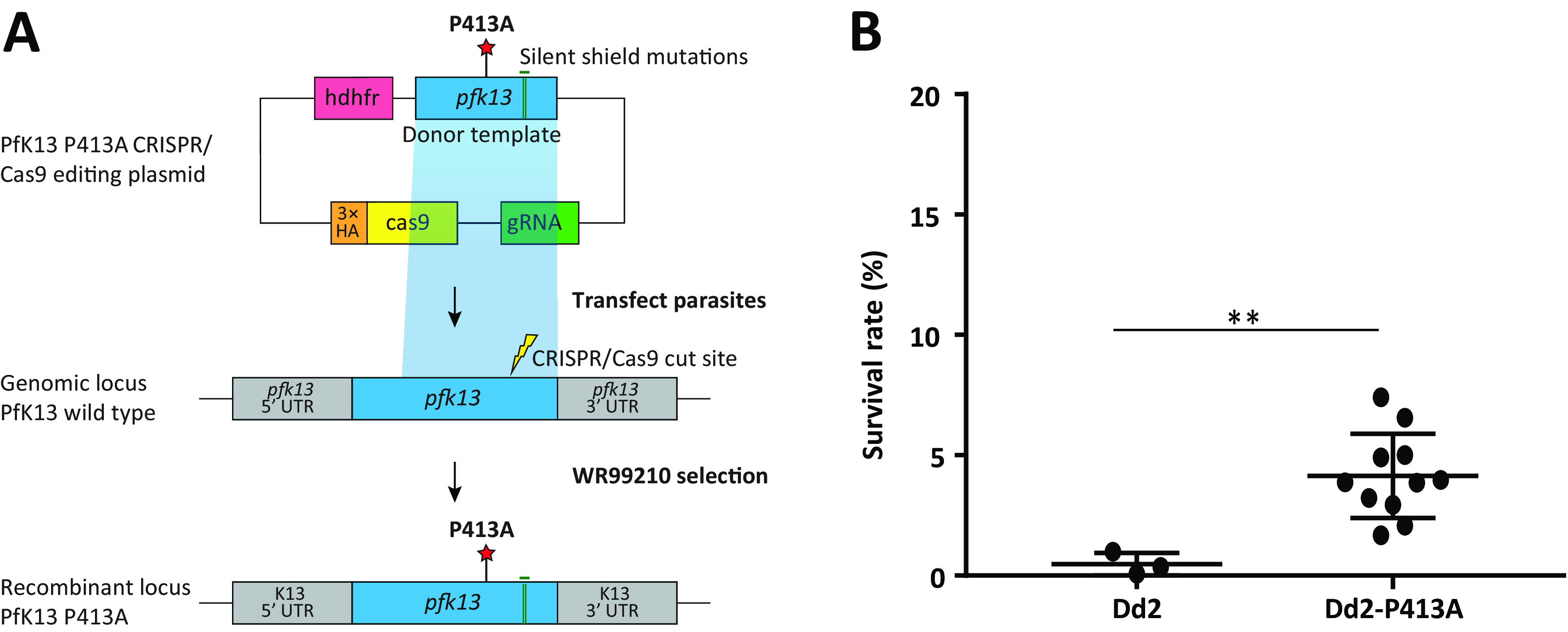
(A) *pfk13* editing strategy. (B) RSA^0-3h^ survival rate of parental and edited Dd2 P. falciparum strains 72 h after exposure to a 6 h pulse of 700 nM DHA. Dd2-P413A groups the three clones Dd2-C8^P413A^/D8^P413A^/F3^P413A^. Mean survival rates and numbers of independent experiments for each strain are presented in supplemental Table S1. *P* values were derived by using the Mann-Whitney *U* test, comparing edited strains to the parental strain. ** *P < *0.01.

To demonstrate the role of the P413A mutation in the ART resistance phenotype observed in the selected lineage, we performed CRISPR/Cas9 gene editing of the ART-sensitive P. falciparum laboratory strain Dd2 (Fig. 2A). Clones were obtained by limiting dilution of the bulk transfectant culture, and the presence of the P413A mutation was verified by Sanger sequencing, leading to the isolation of nine independent P413A mutant clones (Fig. S3). We chose three of these, denoted Dd2-C8^P413A^, Dd2-D8^P413A^, and Dd2-F3^P413A^, and assessed their ART sensitivity using the RSA^0-3h^. The three edited mutant clones exhibited mean RSA^0-3h^ survival rates of 4.2% (Dd2-C8^P413A^), 3.5% (Dd2-D8^P413A^), and 4.7% (Dd2-F3^P413A^), which together were significantly higher than the survival rate of the parental Dd2 strain (0.5%; *P* value = 5.5 × 10^−3^, Mann-Whitney *U* test; Fig. 2B and Table S2). No changes in IC_50_ values for ART were observed between the Dd2 parent and the edited lines (*P* value = 0.321; Table S3). Whole-genome sequencing of the edited Dd2-D8^P413A^ clone (randomly chosen) and the parental Dd2 strain showed monoclonality of both strains, no difference in gene copy number, and 23 non-synonymous SNPs located on 15 different genes (Fig. S1). The P413A mutation was the only SNP also found in the ART-resistant line SMT010p19 (Fig. S4, Table S1 and S4, File S1). The Dd2-D8^P413A^ edited line also carried two synonymous SNPs in the *pfk13* gene (T573T and P574P; File S1).

### Structural and evolutionary characteristics of amino acid position 413 of PfK13.

**FIG 3:**
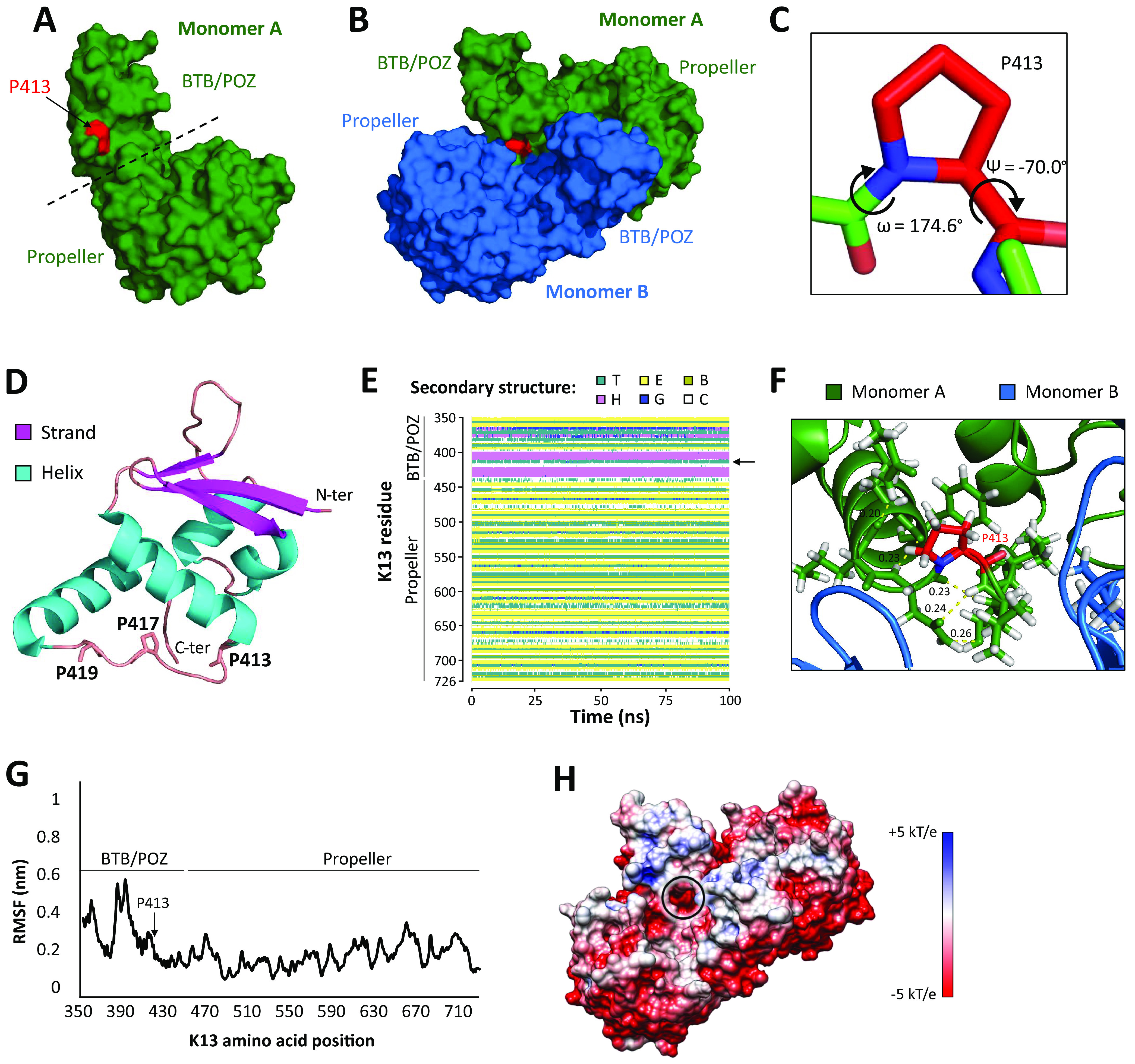
(A) Location of residue P413 on the PfK13 BTB/POZ-propeller structure. The structure is shown as surface, and residue P413 is colored in red. (B) Location of residue P413 on a dimeric structure of PfK13 BTB/POZ-Propeller structure. The structure is shown as surface; monomer A is colored in green, monomer B is colored in blue, and P413 is highlighted in red. (C) Torsion angles of residue P413. (D) Location of P413 on the PfK13 BTB/POZ structure. The structure is colored according to secondary structures. Two other prolines, P417 and P419, are localized on the same structural segment. (E) Evolution of secondary structures for each residue of PfK13 BTB/POZ-propeller structures during 100 ns simulation. The arrow indicates the location of position 413. T, Turn; E, Extended configuration (β-sheet); B, Isolated bridge; H, ɑ-helix; G, 3-10 helix; C, Coil. (F) PfK13 amino acids in contact with P413. Distances are expressed in Angströms. The structure is shown as a cartoon, and amino acids close to P413 are sticks. P413 is colored red and labeled. (G) Cɑ-based root mean square fluctuations (RMSFs) at each residue of the PfK13 BTB/POZ-propeller structure. RMSF values were calculated from the 100 ns molecular dynamics simulations. The arrow indicates the location of the residue 413. (H) Electrostatic surface potential of the homodimeric PfK13 BTB/POZ-Propeller structure, estimated with the APBS method. Electrostatic potential values are in units of kT/e at 298 K, on a scale of −5 kT/e (red) to +5 kT/e (blue). White color indicates a neutral potential.

Based on the PfK13 BTB/POZ-propeller dimeric crystal structure (PDB ID: 4zgc), residue P413 is located in the BTB/POZ domain (Fig. 3A), is exposed at the protein surface of each of the two monomers (accessible molecular surface of P413 = 36.1%; Fig. 3B), and adopts a *cis*-conformation (torsion angles phi = −70.0°, omega = 174.6°; Fig. 3C). Also, P413 is part of a short and proline-rich loop connecting two α-helices (Fig. 3D), and forms a turn at the extremity of one of the helices (Fig. 3D). The P413-induced turn was found to be structurally conserved by molecular dynamics simulation over 100 ns (Fig. 3E). The molecular simulation revealed that P413 was not involved in intramolecular hydrogen bonds (Fig. 3F), was not highly mobile (as expected considering the rigidity of prolines in proteins; RMSF = 0.26 nm; Fig. 3G), and was localized to a region with a very electronegative surface potential at the end of the simulation (Fig. 3H). We then attempted to assess the effect of proline to alanine mutation at position 413 by *in silico* site-directed mutagenesis followed by molecular dynamics simulations. However, the turn initially induced by the wild-type P413 was systematically conserved during the three simulation replicates with the mutant P413A structure in an unrestrained system over 100 ns. We thus could not determine, with confidence, the *in silico* structural impact of P413A mutation from an initial wild-type structure (File S2), because the starting conformation of the P413A mutant structure may likely be folded differently. At the sequence level, the P413A mutation was predicted to have a destabilizing effect on protein stability with a folding free energy change (ΔΔG) of –1.53 as calculated by the machine learning-based SAAFEC-SEQ algorithm, suggesting that P413A has an impact on PfK13 protein structure during the folding process. Finally, the importance of residue P413 for PfK13 protein structure and/or function is reinforced by its full conservation across 35 apicomplexan parasites (*Plasmodium*, *Toxoplasma*, *Eimeria*, *Babesia*, *Neospora*, and *Hammondia*; Table S5).

## DISCUSSION

To study P. falciparum resistance to ART in an African genetic background, we applied a dose-escalating ART regimen over a 1-year period to the ART-sensitive isolate SMT010, collected in the village of Kolle, in Mali, West Africa (25). This strategy was previously and successfully used to identify ART resistance mutations in the propeller domain of *pfk13* and *pfcoronin* from Tanzanian and Senegalese strains, respectively (15, 20). Here, we succeeded in selecting parasite lines with reduced susceptibility to DHA as confirmed by significantly elevated RSA^0-3h^ survival rates in the selected lines relative to the parental line (Fig. 1C and Table S2), with no change in ART IC_50_ values, in accordance with prior studies (Table S3).

Interestingly, ART resistance arose in the strain SMT010 in the same range of ART pressure (between 0.5 μM and 1 μM) as was used earlier to select for the M476I mutation in the F32-ART strain (15). We note that these drug concentrations are within the range of the Cmax achieved in the plasma of patients treated with an ACT. Whole-genome sequencing of the ART-resistant lineage SMT010p19 revealed the acquisition of 11 non-synonymous mutations across seven genes relative to the parental strain SMT010 (Table 1, Fig. S2): the P413A mutation in the *pfk13* gene, and six other mutations not previously related to ART resistance but whose potential role in augmenting or modulating resistance warrants further investigations.

Using CRISPR/Cas9 methodology, we confirmed the ability of the P413A mutation to mediate ART resistance in the ART-sensitive strain Dd2. For this experiment, we generated Dd2-P413A clones that had significantly elevated survival rates in the RSA^0-3h^ (Fig. 2 and Table S2). Only the P413A mutation was common to the ART-resistant SMT010p19 and Dd2-D8^P413A^ parasites (Fig. S4). Consequently, we concluded that the P413A mutation in the *pfk13* gene is sufficient to confer ART resistance *in vitro*. The differences observed in RSA^0-3h^ values between Dd2-P413A clones and SMT010-P413A selected strains (*P* value = 1.5 × 10^−3^, Mann-Whitney *U* test) are consistent with the importance of the genetic background in modulating ART resistance, as previously reported (15, 27). To further explore the role of the genetic background in ART resistance, we looked at SNPs in the two strains used for this study, SMT010 and Dd2, focusing on i) non-synonymous SNPs associated with antimalarial drug resistance; ii) non-synonymous SNPs potentially associated with ART resistance acquisition in a genome-wide association study (28), and iii) non-synonymous SNPs in genes whose inactivation or disruption has been previously associated with ART resistance (29) (Table S6). Of note, the *pfk13* P413A mutation arose under ART pressure in the specific genomic background of the SMT010 strain. As revealed by whole-genome sequencing analysis, SMT010 contains several SNPs related to drug resistance, including in the *mdr1* gene (N86Y and Y184F) that modulates parasite susceptibility to chloroquine, amodiaquine, lumefantrine, mefloquine, and piperaquine *in vitro* (30, 31), and in the *mdr2* gene (F423Y) associated with pyrimethamine resistance (32). Also, SMT010 carries 17 out of the 225 SNPs that were previously shown to foster the acquisition of ART resistance in Cambodian parasite isolates (28). Lastly, SMT010 carries several non-synonymous SNPs in the *mca2*, *kic5*, *eps15*, and *ubp1* genes described as potential K13 interactors (29). The role of these specific mutations in ART resistance has so far not been tested, but disruption or inactivation of these genes was reported to be associated with reduced ART sensitivity (29) (Table S6). When focusing only on these genes, the Dd2 strain presents a different profile, including some SNPs not found in SMT010 (for example, in *crt*, *dhps*, *dhfr*, *fd*, *fp2a*, and *atg18*) and others that were present in both strains like in *mdr2*, *eps15*, or *tRNA pseudouridine synthase* (Table S6). The role of these genes in the acquisition and/or expression of *in vitro* ART resistance also remains elusive.

Remarkably, the P413A mutation is located in the BTB/POZ domain of PfK13 (amino acids 350–437) (16). To date, only 17 non-synonymous mutations located in the BTB/POZ domain of PfK13 have been reported in clinical isolates and only one, D353Y (found in five isolates from Vietnam), was associated with a parasite clearance half-life (PCt_1/2_) > 5 h after ACT treatment (Table S7) (23, 24, 33–40). Neither P413A nor other missense mutations at position 413 have been reported so far, whereas the synonymous mutation P413P (codon CCC → CCG or CCT) was reported in 12 African and Asian isolates (23). To date, there are too few data available from selection experiments or epidemiological surveillance studies to hypothesize whether SNPs in the K13 BTB/POZ domain would be more frequently observed in African genetic background. The reasons why P413A has not yet emerged in the field are unclear. This could be related to genetic background issues modulating the level of ART resistance or parasite fitness, or an effect of the P413A mutation on some essential function of PfK13 during the parasite life cycle. Importantly, while molecular epidemiology studies of *pfk13* are often limited to the propeller domain, here we provide compelling evidence that a mutation outside the propeller domain can confer ART resistance *in vitro*. We note that one *pfk13* mutation located out of the propeller domain, the E252Q mutation located in the coiled-coil, was especially successful in the early evolution of *pfk13* mutations in parasites from the Thai-Myanmar border and until the C580Y mutation took over. E252Q was confirmed to significantly increase parasite survival rate *in vivo* on at least one Thai genetic background (21, 27, 41).

In proteins homologous to PfK13 such as the KEAP1 protein, whose dysfunction is involved in certain cancers, the BTB domain mediates varying oligomerization architectures and interacts with the cullin-3 ubiquitin ligase. In contrast, the propeller domain often serves as a receptor for substrate proteins to be ubiquitinated (42, 43). A 3D structure of the entire PfK13 protein is still lacking, however a structure is available for a truncated protein consisting of the two C-terminal domains, namely, the BTB/POZ and propeller domains (https://www.rcsb.org/structure/4ZGC). The truncated protein is solved as a homo-dimer, but the entire protein might have a more complex oligomeric organization with intra- and inter-molecular interactions not shown by the currently available structure. With this limitation in mind, the evolutionary conserved P413 position appears to be exposed at the protein surface of the two monomers. We can thus hypothesize that the P413A mutation may disturb the formation of proper PfK13 oligomeric states or some inter-molecular interactions. P413 is also located close to two other evolutionarily conserved prolines, P417 and P419, on a short coil region connecting two α-helices (Fig. 3D). The cyclic structure of proline's side chain induces exceptional conformational rigidity (44). Such a rigidity may be reduced in the P413A mutant structure, since the turn normally formed by P413 may likely be lost during the folding process. Molecular dynamics simulations on the *in silico*-generated P413A mutant structure did not reveal any changes in stability, probably because the starting structure was not a reflection of the real mutant structure. Rather, a machine learning-based algorithm at the sequence level revealed that P413A mutation has a destabilizing effect on protein stability. Such an effect could result in lower PfK13 abundance, a known driver of ART resistance in R539T and C580Y mutants (29, 45, 46).

### Conclusion.

Mutation of BTB/POZ domain of the PfK13 protein is here shown to be associated with *in vitro* ART resistance. This observation was first made in a P. falciparum lineage obtained from an African isolate subjected to *in vitro* ART pressure and was then validated by gene editing.

While monitoring of ART resistance is often limited to the *pfk13* propeller domain, here we provide compelling evidence that a mutation in the BTB/POZ domain can also confer ART resistance.

## MATERIALS AND METHODS

### P. falciparum culture.

Two P. falciparum strains were used: the field isolate SMT010 collected in Mali (25), and the Dd2 laboratory strain. Parasites were cultured according to the Trager and Jensen method with slight modifications (47). The field isolate and its derived lineages were cultured at 2% hematocrit in type O human red blood cells (EFS, French Blood Bank) in RMPI-1640 medium supplemented with 5% human serum (EFS, French Blood Bank), 0.055% hypoxanthine, 5.5% Albumax II (Thermo Fisher), 1 mM l-glutamine (Merck), and 11 μg/ml gentamicin (Merck), at 37°C with 5% O_2_, 5% CO_2_, and 90% N_2_. The laboratory strain was cultivated at 2% hematocrit in type O human red blood cells in RMPI-1640 medium supplemented with 5% human serum, at 37°C with 5% CO_2_ in a humidified atmosphere.

### Drugs.

Artemisinin (ART) was purchased from TCI Europe, dihydroartemisinin (DHA) was provided by WWARN, and WR99210 and ampicillin were purchased from Merck.

### Selection of ART resistance.

The ART-sensitive field isolate SMT010 was submitted to a drug pressure protocol adapted from the one that allowed for the selection of the ART-resistant strain F32-ART (14). Briefly, d-sorbitol synchronized ring-stage parasites at 3% parasitemia in culture medium supplemented with 10% human serum were exposed to increasing doses of ART for 24 h. After drug exposure, the parasite culture was washed with RPMI 1640 medium and returned to drug-free culture conditions (at 10% human serum) in a new flask. Parasitemia was microscopically monitored by Giemsa-stained thin blood smears until the culture reached 3% parasitemia. Then the parasite culture was placed in normal culture conditions (5% human serum) until the next drug pressure cycle. Twenty-four cycles of drug pressure were done over 1 year: 1 to 3 drug pressure cycles with ART doses ranging from 10 nM to 750 nM, and from 1 μM dose parasite cultures were exposed to 3 drug pressure cycles at each dose (Fig. 1A and B).

### Ring-stage survival assay.

The Ring-Stage Survival Assay (RSA^0-3h^) was carried out as previously reported (13). Briefly, 0–3 h ring-stage parasites synchronized by percoll/sorbitol treatment were exposed in duplicate in 48-well plates to 700 nM DHA or 1% DMSO for 6 h, then washed in RPMI 1640 and returned to normal culture conditions for the next 72 h. Survival rates were measured microscopically by Giemsa-stained thin blood smears. Parasitemia was calculated by counting 10,000 red blood cells (RBCs) per condition by two independent microscopists. Assays with a less than 2-fold parasite replication rate across the 72 h time frame of the experiment in DMSO control cultures were discarded. A survival rate of DHA-treated parasites relative to control above 1% in RSA^0-3h^ indicated ART resistance (48). Statistical analyses of the data were done using GraphPad Prism software.

### Standard chemosensitivity assay.

The antimalarial activity of ART was evaluated using the SYBR green I method (49). This assay was carried out in 96-well culture plates on d-sorbitol synchronized ring-stage parasites at 1% parasitemia (50). Drug testing was performed in triplicates in a dose range of 5 concentrations. The parasites were incubated with the drugs for 48 h. Parasite pellets were then washed in 1× PBS in order to reduce background noise during the plate reading, prior to lysing RBCs at −20°C overnight. Then, the plates were thawed and 100 μl from each well was transferred into a black 96-well plate. One hundred μl per well of SYBR green I (Thermo Fisher) diluted at a final concentration of 2× in lysis buffer was added and samples were left to incubate for 1 h at room temperature prior to reading the plates on BioTek FLx800 Microplate Fluorescence Reader (λ_excitation_ = 485 nm, λ_emission_ = 528 nm). IC_50_ determination and statistical analysis of these data were done using GraphPad Prism software.

### Sanger sequencing.

Parasite DNA was extracted with the High Pure PCR Template Preparation Kit (Roche Diagnostic) according to the manufacturer’s instructions. The *pfk13* gene was amplified by PCR using the published primer set (Fw – GGGAATCTGGTGGTAACAGC/Rev – CGGAGTGACCAAATCTGGGA) (https://www.wwarn.org/tools-resources/procedures/pcr-and-sequencing-genotyping-candidate-plasmodium-falciparum-artemisinin) and the DreamTaq Hot Start DNA polymerase (Invitrogen) with the following parameters: initial denaturation 95°C 15 min, denaturation 95°C 30 s, annealing 58°C 1 min, extension 72°C 2 min, 30 X, final extension 72°C 10 min. Sanger sequencing of the *pfk13* gene was performed by the Genoscreen Company (France) with the following primers: Fw – GGGAATCTGGTGGTAACAGC, Fw –GCCTTGTTGAAAGAAGCAGA, Rev – CGCCAGCATTGTTGACTAAT, Rev – CGGAGTGACCAAATCTGGGA. Sequences were compared to the reference genome of P. falciparum 3D7 strain by BLASTN using GeneDB (https://www.genedb.org) to identify SNPs.

### CRISPR/Cas9 plasmid construction.

Modification of the *pfk13* locus to introduce the P413A mutation was performed by CRISPR/Cas9 editing using the pDC2-cam-coSpCas9-U6-gRNA-hdhfr all-in-one plasmid that contains a P. falciparum codon-optimized Cas9 sequence, a dihydrofolate reductase (*dhfr*) expression cassette conferring resistance to WR99210, and restriction enzyme sites for insertion of a guide RNA (gRNA) and donor template. A *pfk13*-specific guide RNA (gRNA) was introduced into this vector at the BbsI restriction sites by T4 DNA ligase (NEB) using the following primer pair: Fw – TATTACACATAGCTGATGATCTAG, Rev – AAACCTAGATCATCAGCTATGTGT. Primers were phosphorylated and annealed prior to ligation. A donor template consisting of an ∼1.5 kb region of the *pfk13* coding region was amplified using the primer pair Fw – GTGACGTCGATTGATATTAATGTTGGTGGAGC and Rev – CCGCATATGGTGCAAACGGAGTGACCAAATCTGGG, and cloned into the pGEM T-easy vector system (Promega). This donor sequence was subjected to site-directed mutagenesis in the pGEM vector to introduce the *pfk13* P413A mutation as well as silent shield mutations at the Cas9 cleavage site using the following primer pairs: Fw – CTTAACTTCTTAAGAAATGCGTTAACTATACCCATACCAAAAGATTTAAGTGAAAGTG and Rev – GGTATGGGTATAGTTAACGCATTTCTTAAGAAGTTAAGTATAATTCTAAATAACTC, and Fw – GAATACGCCAAGATCATCAGCTATGTGTGTTGCTTTTGATAATAAAATTTATGTCATTGG and Rev – GCAACACACATAGCTGATGATCTTGGCGTATTCAAAGGTGCCACCTCTACCC, respectively. Finally, the *pfk13* donor sequence was amplified from the pGEM vector using the primer pair Fw – GAGGTACCGAGCTCGAATTCGAAACGGAATTAAGTGATGCTAG and Rev – CGAAAAGTGCCACCTGACGTCAAACGGAGTGACCAAATCTGGG, and subcloned into the pDC2-cam-coSpCas9-U6-gRNA-hdhfr plasmid at the EcoRI and AatII restriction sites by In-Fusion Cloning (TaKaRa).

### Plasmid amplification.

The CRISPR/Cas9 plasmid was amplified in DH5α competent cells (Invitrogen). Briefly, competent cells were transformed by thermic shock (successive incubations: 15 min on ice, 45 s at 42°C, 2 min on ice) prior to spreading over LB agar petri dishes with 50 μg/ml ampicillin and incubating at 37°C overnight. Transformed colonies were then grown in 200 ml LB medium with 50 μg/ml ampicillin at 37°C overnight with shaking. Plasmids were purified using the plasmid Midiprep kit (Qiagen) according to the manufacturer’s instructions and resuspended in 40 μl DNase free water + 260 μl of Cytomix solution (51). Plasmid concentration was adjusted to 0.2 μg/μL in a final volume of 220 μl of Cytomix prior to electroporation.

### Parasite transfection and selection of clones.

The P. falciparum Dd2 strain was used for transfection experiments. Briefly, ring-stage parasites were electroporated with the purified plasmid as previously described (52) using the Gene Pulser X cell (Bio-Rad) with the following parameters: exponential time protocol Volt 301/Cap 950/Ω∞/1 pulse. Then 24 h later parasites were exposed to 2.5 nM WR99210 to select for transformed parasites until parasites became microscopically detectable. DNA from bulk cultures was extracted and P413A *pfk13* editing was assessed by Sanger sequencing as detailed above. Cultures showing the expected mutation were selected for 48-well cloning by limiting dilution. Clone proliferation was monitored microscopically, and *pfk13* gene editing was assessed by DNA extraction, PCR, and Sanger sequencing as detailed above.

### Whole-genome sequencing, assembly, and analyses.

Whole-genome sequencing was performed on Dd2, Dd2-D8^P413A^, SMT010, and SMT010p19 using Illumina NextSeq 500 technology. Prior to library preparation, total DNA was quantified using the Qubit dsDNA high sensitivity kit (Thermo Fisher Scientific) according to the manufacturer’s recommendations. PCR-free Illumina libraries were prepared using KAPA Hyper Prep Kit (Roche), and mechanical DNA shearing was performed through microTUBE-50 AFA Fiber Screw-Cap (Covaris) using a setting of 30% duty factor, 100W peak incidence power, and 1000 cycles per burst for 150 s. DNA libraries were sequenced at the Cochin Institute, GENOM’IC platform (Paris, France) for 150 bp paired-end reads.

Sequence data obtained from each sample were subjected to standard Illumina QC procedures, and reads were checked for quality, content, and coverage (Table S8). Reads were mapped to the P. falciparum 3D7 reference genome (PlasmoDB, release 39, https://plasmodb.org/) using bwa-mem software (53). Mean coverage, percentage genome covered with at least 10 reads, and GC content were calculated using Qualimap v2.2.1 (54). Prior to variant calling, read sequences were sorted, and duplicate reads were removed using the Picard tool MarkDuplicates (55). Potential SNPs were discovered by running GATK (version 4.1.8) tool HaplotypeCaller independently across each of the samples with default parameters (55). SNPs from each sample were then merged using the GATK tool CombineGVCFs, and genotyped using the GATK tool GenotypeGVCFs. SNPs were then subjected to several filtering steps. First, an SNP that was not covered by at least 5 reads was removed using VCFtools. Second, hard filtering was done to remove potential false positives based on the following filter: QUAL < 30 | QD < 2 | MQ < 40. Finally, only SNPs that were biallelic were retained using GATK SelectVariants and the following parameters: -select- type SNP –restrict-alleles-to BIALLELIC. SNPs were annotated using SnpEff v.4.11 from the P. falciparum 3D7 GFF annotation file using the following options: -no-downstream -no-upstream -onlyProtein (56). Copy number variation was evaluated across the whole-exome of P. falciparum using the custom read depth strategy and PlasmoCNVScan (57). Clonality of the samples was assessed by estimating the *F*_ws_ statistic from variant data using the R package *moimix*. A *F*_ws_ < 0.95 is indicative of a clonal infection.

### Molecular dynamics simulations and structural analyses.

The K13 BTB/POZ-Propeller crystallographic structure was retrieved from the Protein Data Bank (PDB) repository, PDB ID: 4zgc. For the mutant structure, the P413A mutation was introduced using the *swapaa* function of UCSF Chimera by substituting the residue with the most probable rotameric conformation (58). All missing atoms were then added using Swiss PDB Viewer (59). Both wild-type and mutant structures were checked for quality using MolProbity (60), which showed no outliers and placed > 98% of residues (including the *in silico* introduced mutation) in favored regions.

Molecular dynamics simulations were carried out on both monomeric and homodimeric states of BTB-propeller wild-type and mutant structures, using the GROMACS package v. 5.1.5. (61) with the improved side chain torsion potentials force field Amber99ss-ILDN for amino acid interaction (62). Protein systems were immersed in a dodecahedron box of TIP3P water molecules preserving at least 13 Å of separation between the solute and the edges of the box. The Particle Mesh Ewald approach (PME) (63) was employed with van der Waals and Coulomb non-bonded interactions truncated at 10 Å. Bond lengths were constrained using the LINCS algorithm (64) that allowed a 2 femtosecond (fs) time step in all simulations. The ionization state of residues was set to be consistent with neutral pH, and Na^+^ counter-ions were then added by randomly replacing water molecules to ensure the overall charge neutrality of the system. The disulfide bridge between the C532 and C580 residues (distance: 2.07 Å) was considered by editing the specbond file of GROMACS (disulfide bridge distance cutoff set to 2.1 Å). The whole system consisted of ∼80,000 atoms for the monomeric states, and ∼109,000 atoms for the homodimeric states. To release conflicting contacts, solvated systems were subjected to energy minimization using the steepest descent algorithm over 5,000 steps until the maximum force was < 1,000 kJ/mol^−1^/nm^−1^. Before molecular dynamics productions, each solvated system was subjected to two-step equilibration. In the first step, systems were equilibrated for 100 ps in the NVT ensemble at 300 K with the V-rescale temperature coupling (65). The equilibrated systems from the NVT ensemble were then treated to constant pressure (NPT) ensemble for 100 ps using the Parrinello-Rahman barostat (66) under an isothermal-isobaric pressure of 1.0 bar. Position restraints were applied to all atoms during equilibration steps to avoid configuration changes. Molecular dynamics productions were run for 100 ns in the absence of any restraints. During productions, the V-rescale thermostat coupled with the Parrinello-Rahman barostat were used to maintain the temperature and pressure at 300 K and 1.0 bar, respectively. Frames of the trajectories were stored at every 10 ps. Wild-type and mutant systems were subjected to two molecular dynamics replicates with the same parameters and algorithms.

Molecular dynamics trajectories were analyzed using inbuilt GROMACS tools. The first ns of the trajectories, corresponding to equilibration time, was removed prior to analyses. Root mean square deviation (RMSD; *rms* function in GROMACS) and radius of gyration (Rg; *gyrate* function) were calculated on protein backbone heavy atoms to measure protein stability and compactness, respectively. Local changes in atoms were investigated by measuring root mean square fluctuations (RMSF; *rmsf* function) on side chain residues and on Cα atoms. The number of distinct hydrogen bonds (*hbond* function) formed within the protein during simulations was counted when the donor-acceptor distance was ≤ 3.5 Å and donor-hydrogen-acceptor angle ≤ 30°. Additionally, the average intramolecular contacts between each pair of residues formed during the course of molecular dynamics simulations was analyzed through contact mapping using the *mdmat* function. Protein secondary structures throughout the simulations were investigated using the *Timeline plugin* available in VMD (67).

The electrostatic potential was calculated using the Adaptive Poisson-Boltzmann Solver (APBS) method (68). The pqr input file required to run APBS was prepared using PDB2PQR (69). A grid-based method was used to solve the linearized Poisson-Boltzmann equation at 298 K, with protein and solvent dielectric constant values fixed at 2 and 78.5, respectively. The surface potentials were set to lie between ± 5 *kT/e*.

The effect of the P413A mutation on protein thermodynamics stability was assessed by calculating the folding free energy change (ΔΔG) using the machine learning-based SAAFEC-SEQ algorithm at the sequence level.

### Molecular imaging.

All protein structural visualizations were performed using UCSF Chimera (58).

### Epidemiological analysis.

The catalogue of genetic variation in P. falciparum from MalariaGEN P. falciparum community project release 6.0 data (Pf3k Pilot Data Release 6) (23) was used to estimate global diversity in the *pfk13* gene. The catalogue includes 7,113 whole-genome sequences of P. falciparum isolates collected from 73 locations in Africa, Asia, South America, and Oceania between 2002 and 2015. Data on *pfk13* were obtained by downloading the P. falciparum genetic diversity of chromosome 13 (https://www.malariagen.net/data/catalogue-genetic-variation-p-falciparum-v6.0), then extracted by providing the *pfk13* gene location (1,724,817 to 1,726,997) using the *intersect* function of bedtools v2.25.0 (70). A variant was considered when the read depth was ≥ 10, and the proportion of alternate reads compared to reference reads was ≥ 10%.

### Collection of K13 orthologous sequences and sequence alignment.

The amino acid sequence of PfK13 (PF3D7_1343700) was queried against the specialized eukaryotic pathogen database VEuPathDB (release 48) (71) and the NCBI non-redundant protein database using blastp and tblastn searches (BLOSUM62 scoring matrix). Thirty-five K13 sequences were retrieved from distinct 21 *Plasmodium* species and 14 other apicomplexan parasites. Protein sequence alignment was generated using MAFFT version 7 (E-INS-I strategy with BLOSUM62 scoring matrix, gap opening penalty 2.0 and offset 0.1) (72).
