# Supplementary material for: Mutation in the Plasmodium falciparum BTB/POZ Domain of K13 Protein Confers Artemisinin Resistance
Source: Antimicrob Agents Chemother. 2022 Jan 18;66(1):e01320-21. doi: 10.1128/AAC.01320-21 (PMC8765297; doi:10.1128/AAC.01320-21)
Supplement: Supplemental file 2 — Supplemental material. Download AAC.01320-21-s0001.pdf, PDF file, 1.2 MB [file aac.01320-21-s0001.pdf]

Mutation in *Plasmodium falciparum* BTB/POZ domain of K13 protein confers artemisinin resistance

Paloque *et al*.,

Supplemental figures and tables:

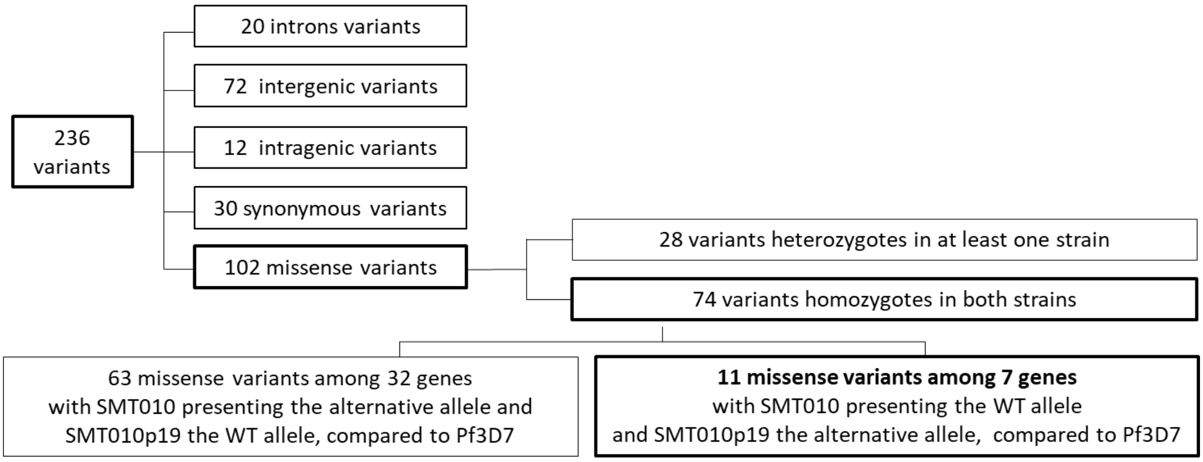

Figure S1: Genetic variations reported between the parental strain SMT010 and the selected ART-resistant line SMT010p19. The sequences were aligned to the genome of the reference strain Pf3D7. The complete list of the 236 variants is available in Supplementary Data (File S1). Among them, 102 variants were missense mutations of which 74 were in a homozygous state. Sixty-three missense mutations were specifically found in SMT010, localized in 32 genes with unknown function or involved in gene regulation and protein expression. SMT010p19 had 11 missense mutations not found in SMT010 and distributed across 7 genes.

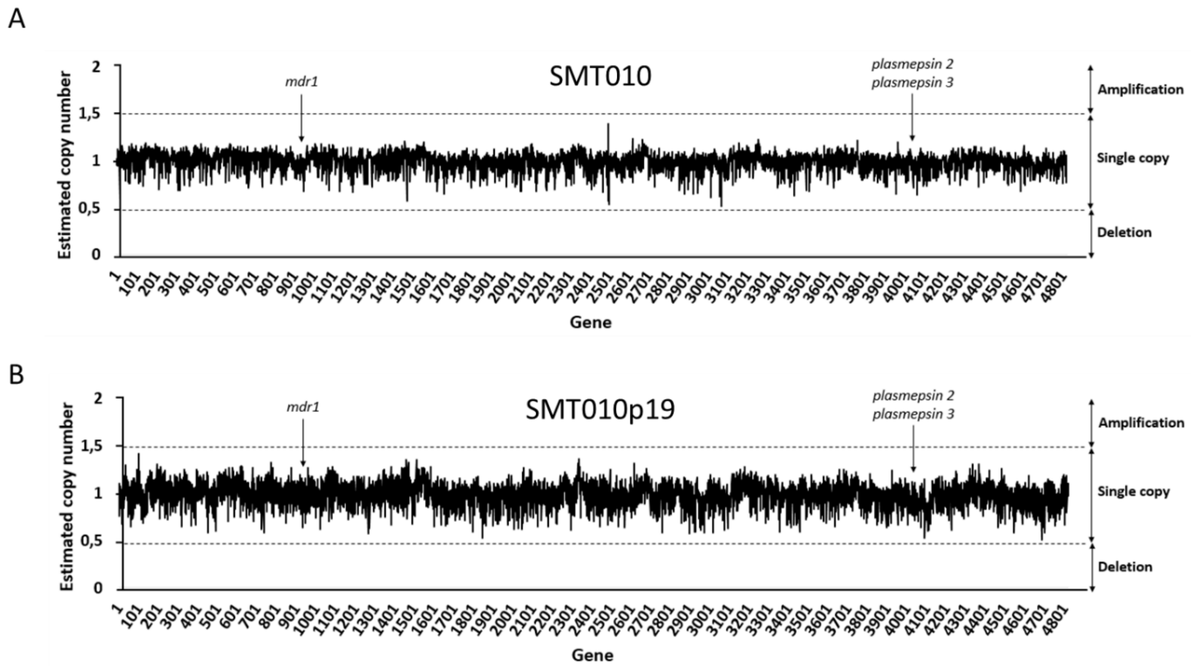

Figure S2: (A) Copy number variation analysis of SMT010. **(B)** Copy number variation analysis of SMT010p19.

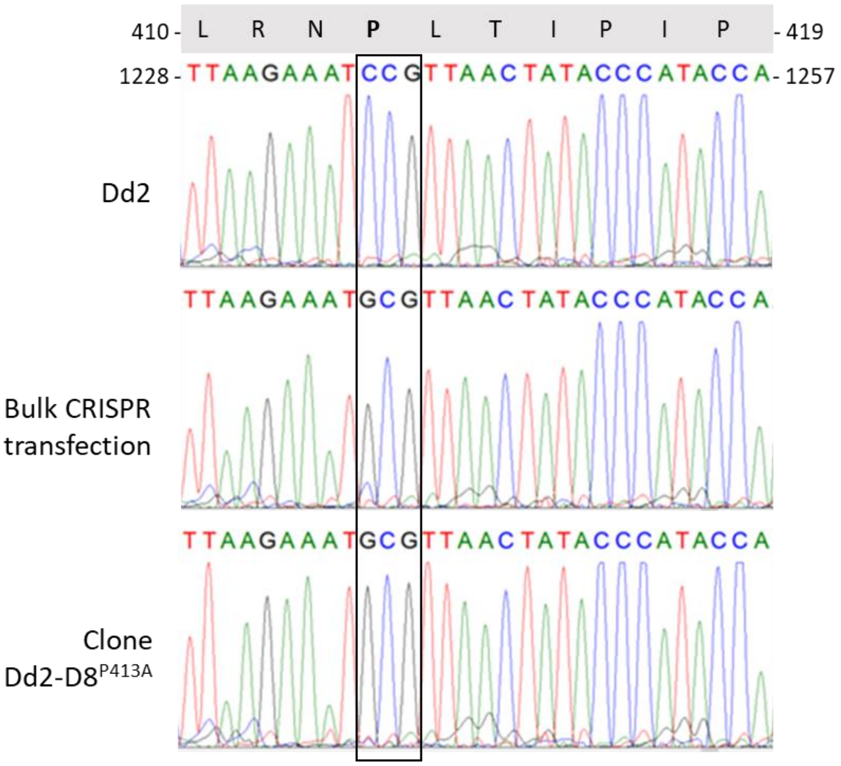

Figure S3: Sanger sequencing of the parental artemisinin-sensitive strain Dd2, the bulk transfection and the edited clone Dd2-D8^{P413A}. The position of codon 413 is framed.

A

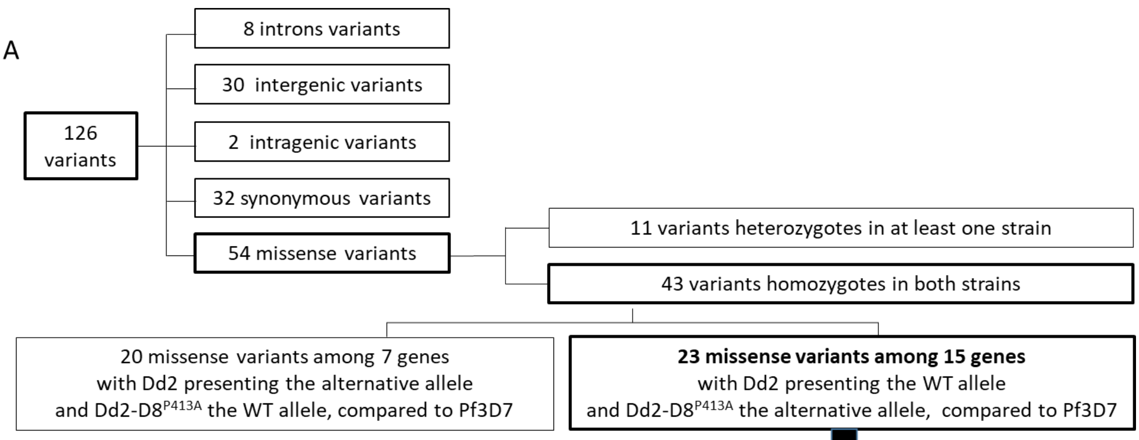

Gene ID	Gene name	SNPs in Dd2-D8^{P413A}
PF3D7_0102200	ring-infected erythrocyte surface antigen	N915H
PF3D7_0307900	conserved Plasmodium protein, unknown function	Q1647Glu
PF3D7_0405300	liver specific protein 2	D1864N, D1865G
PF3D7_0425200	Plasmodium exported protein (hyp15), unknown function	C182S
PF3D7_0501800	chromatin assembly factor 1 subunit A	N802D, D806N, G809D, D813G
PF3D7_0613800	AP2 domain transcription factor	M472I, S473L
PF3D7_0813700	ABC transporter F family member 1	P903Q
PF3D7_1106500	conserved Plasmodium protein, unknown function	E216K
PF3D7_1140700	chromo domain-containing protein	N2192S
PF3D7_1141100	conserved Plasmodium protein, unknown function	S137L
PF3D7_1245200	conserved Plasmodium membrane protein, unknown function	I602V
**PF3D7_1343700**	**kelch protein K13**	**P413A**
PF3D7_1366400	rhoptry protein RHOP148	V269I, V269A
PF3D7_1415400	Btz domain-containing protein, putative	R797H, R799K, N802K
PF3D7_1442600	TRAP-like protein	D1460H

B

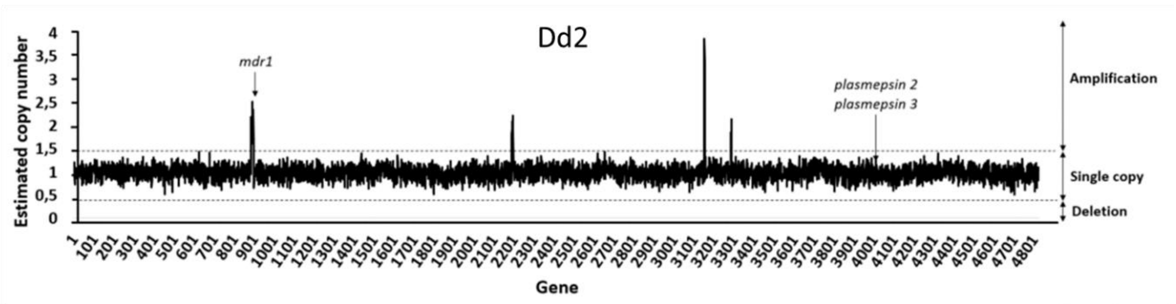

C

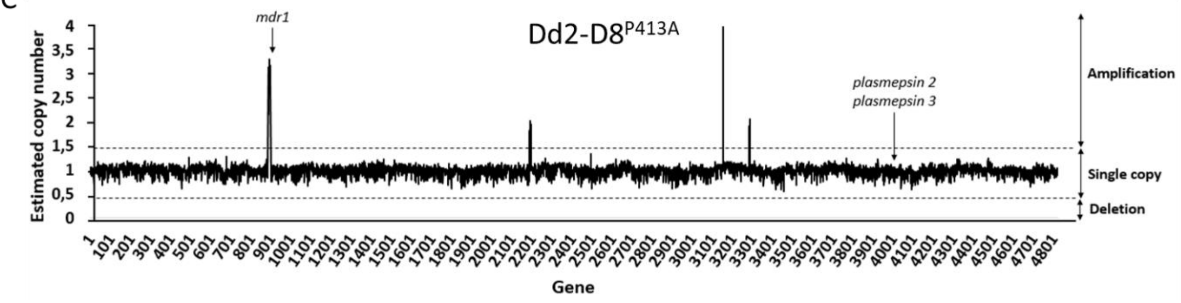

Figure S4: (A) Genetic variations reported between the strain Dd2 and the edited strain Dd2-D8^{P413A}. The sequences were aligned to the genome of the reference strain Pf3D7. The complete list of the 126 variants is available in Supplementary Data (File S1) This analysis confirmed the presence of the P413A mutation in the *pfk13* gene and revealed 125 other genetic variations including two synonymous SNPs (T573T and P574P) in the *pfk13* gene and 22 non-synonymous SNPs among 14 genes (File S1). **(B)** Copy number variation analysis of Dd2. **(C)** Copy number variation analysis of Dd2-D8^{P413A}. No difference was detected between both strains and the list of genes amplified in Dd2 and Dd2-D8^{P413A} strains is available in Table S5.

Table S1: Assessment of clonality for the two *P. falciparum* strains and their corresponding selected/edited lines subjected to whole-genome sequencing.

**Sample**	**F_{ws} statistic** [a]	**Clonality**
SMT010	0.986	Monoclonal
SMT010p19	0.973	Monoclonal
Dd2	0.982	Monoclonal
Dd2-D8^{P413A}	0.983	Monoclonal

Note – [a] Clonality of the samples was assessed by estimating the F_{ws} statistic from variant data using the package R *moimix*. A F_{ws} > 0.95 is indicative of a monoclonal infection.

Table S2: RSA$^{0\text{-}3h}$ survival rate 72 h after exposition to a 6-h pulse of dihydroartemisinin 700 nM of the *P. falciparum* strains Dd2, Dd2-P413A (including different Dd2 clones after gene editing), SMT010 and SMT010-P413A (including different SMT010 lineages after artemisinin resistance acquisition).

**Mean Survival rate ± SEM (%) of SMT010 lineages**									
WT	Selected (WT)		*p* value$ (WT vs SMT010p15)	Selected (P413A)					*p* value$ (WT vs Selected)
SMT010	SMT010p9	SMT010p15		SMT010p18	SMT010p19	SMT010p20	SMT010p21	SMT010p24	
0.4 ± 0.2	0.24	2.5 ± 0.6	0.057	9.2 ± 0.7	9.6 ± 2.4	11.6	6.9 ± 2.6	10.7	0.0008

**Mean Survival rate ± SEM (%) of Dd2 laboratory line**				
WT	Edited (P413A)			*p* value$ (WT vs Edited)
Dd2	Dd2-C8^{P413A}	Dd2-D8^{P413A}	Dd2-F3^{P413A}	
0.5 ± 0.2	4.2 ± 0.4	3.5 ± 1.6	4.7 ± 0.7	0.0055

$Mann-Whitney *U* test. A *p* value < 0.05 was considered as statistically significant.

Results represent the mean: *i*) of three independent experiments for SMT010p15, SMT010p18, Dd2 and Dd2-C8^{P413A}, and *ii*) of four independent experiments for SMT010, SMT010p19, SMT010p21, Dd2-D8^{P413A} and Dd2-F3^{P413A}, each one performed in duplicate. Smears reading was assessed independently by two microscopists.

Table S3: Evaluation of chemo-sensitivity to artemisinin (ART) by standard assay of *P. falciparum* strains Dd2, Dd2-P413A (including different Dd2 clones after gene editing), SMT010 and SMT010-P413A (including different SMT010 lineages after ART resistance selection).

	Mean IC_{50} values ± SEM (nM) of SMT010 lineages					Mean IC_{50} values ± SEM (nM) of Dd2 laboratory line				
	WT	Selected (P413A)			*p* value$ (WT vs Selected)	WT	Edited (P413A)			*p* value$ (WT vs Edited)
	SMT010	SMT010p19	SMT010p21	SMT010p24		Dd2	Dd2-C8^{P413A}	Dd2-D8^{P413A}	Dd2-F3^{P413A}	
ART	1.8 ± 0.3	5.7 ± 1.9	5.5 ± 3.5	7 ± 0.7	0.107	11.3 ± 0.3	28.5 ± 7.5	13.3 ± 1.8	11.9 ± 0.9	0.321

$Mann-Whitney *U* test. A *p* value < 0.05 was considered as statistically significant.

Results represent the mean of two independent experiments.

Table S4: List of genes amplified in Dd2 and Dd2-D8^{P413A} strains.

Gene ID	Gene name
PF3D7_0521900	conserved Plasmodium protein, unknown function
PF3D7_0522000	conserved Plasmodium protein, unknown function
PF3D7_0522100	conserved Plasmodium protein, unknown function
PF3D7_0522300	18S rRNA (guanine-N(7))-methyltransferase, putative
PF3D7_0522400	conserved Plasmodium protein, unknown function
PF3D7_0522500	50S ribosomal protein L17, apicoplast, putative (rpl17)
PF3D7_0522600	inner membrane complex protein
PF3D7_0522700	iron-sulfur assembly protein (SufA)
PF3D7_0522800	pre-mRNA-splicing factor BUD31, putative (bud31)
PF3D7_0522900	zinc finger protein putative
PF3D7_0523000	multidrug resistance protein 1 (mdr1)
PF3D7_0523100	mitochondrial-processing peptidase subunit alpha, putative
PF3D7_0523200	conserved Plasmodium protein, unknown function
PF3D7_1007100	conserved Plasmodium protein, unknown function
PF3D7_1007200	rho GTPase-activating protein, putative
PF3D7_1007300	RING zinc finger protein, putative
PF3D7_1007400	conserved Plasmodium protein, unknown function
PF3D7_1007500	conserved Plasmodium protein, unknown function
PF3D7_1007600	conserved Plasmodium protein, unknown function
PF3D7_1007700	AP2 domain transcription factor AP2-I (ap2-i)
PF3D7_1007800	conserved Plasmodium protein, unknown function
PF3D7_1223800	citrate/oxoglutarate carrier protein, putative (yhm2)
PF3D7_1223900	50S ribosomal protein L24, putative
PF3D7_1224000	GTP cyclohydrolase I (gch1)
PF3D7_1237200	conserved Plasmodium protein, unknown function
PF3D7_1237300	conserved Plasmodium protein, unknown function
PF3D7_1237400	conserved Plasmodium protein, unknown function
PF3D7_1237500	conserved Plasmodium protein, unknown function

Table S5: List of K13 orthologous sequences and conservation of proline (P) in position 413 among 21 *Plasmodium* species and 14 other apicomplexan parasites.

**Species**	**Accession number** [a]	**Source**	**Amino acid at position 413**
Plasmodium falciparum	PF3D7_1343700	PlasmoDB	P
Plasmodium reichenowi	XP_012764805.1	Genbank (NCBI)	P
Plasmodium gaboni	XP_018640212.1	Genbank (NCBI)	P
Plasmodium vivax	XP_001614215.1	Genbank (NCBI)	P
Plasmodium inui	XP_008815744.1	Genbank (NCBI)	P
Plasmodium knowlesi	XP_002259918.1	Genbank (NCBI)	P
Plasmodium fragile	XP_012337473.1	Genbank (NCBI)	P
Plasmodium cynomolgi	PCYB_122000	PlasmoDB	P
Plasmodium vinckei vinckei	XP_008622491.1	Genbank (NCBI)	P
Plasmodium vinckei petteri	EUD71747.1	Genbank (NCBI)	P
Plasmodium chabaudi chabaudi	CDR16038.1	Genbank (NCBI)	P
Plasmodium chabaudi adami	SCM11204.1	Genbank (NCBI)	P
Plasmodium yoelii	XP_730901.1	Genbank (NCBI)	P
Plasmodium berghei	CDS51002.1	Genbank (NCBI)	P
Plasmodium coatneyi	PCOAH_00041530	PlasmoDB	P
Plasmodium gallinaceum	PGAL8A_00254800	PlasmoDB	P
Plasmodium relictum	PRELSG_1254200	PlasmoDB	P
Plasmodium gonderi	GAW82138.1	Genbank (NCBI)	P
Plasmodium malariae	PmUG01_12021200	PlasmoDB	P
Plasmodium ovale wallikeri	ANG83523.1	Genbank (NCBI)	P
Plasmodium ovale curtisi	PocGH01_12019400	PlasmoDB	P
Toxoplasma gondii	ESS31969.1	Genbank (NCBI)	P
Hammondia hammondi	XP_008882882.1	Genbank (NCBI)	P
Neospora caninum	XP_003882792.1	Genbank (NCBI)	P
Eimeria acervulina	XP_013247290.1	Genbank (NCBI)	P
Eimeria falciformis	EfaB_MINUS_11414	ToxoDB	P
Eimeria brunetti	CDJ54128.1	Genbank (NCBI)	P
Eimeria maxima	XP_013334533.1	Genbank (NCBI)	P
Babesia microti	XP_012648586.1	Genbank (NCBI)	P
Babesia bovis	XP_001609009.1	Genbank (NCBI)	P
Babesia bigemina	tann.chr01	GeneDB	P
Theileria parva	XP_766008.1	Genbank (NCBI)	P
Theileria equi	XP_004829715.1	Genbank (NCBI)	P
Theileria orientalis	XP_009689263.1	Genbank (NCBI)	P
Theileria annulata	XP_012767816.1	Genbank (NCBI)	P

[a] Identifier of the protein sequence retrieved from sequence databases.

Table S6: Genetic background concerning previously described non-synonymous SNPs associated with antimalarial drug resistance of the *P. falciparum* artemisinin-sensitive strains SMT010 and Dd2 (*vs* the reference strain 3D7). The complete list of genetic variations present in both strains are available in Supplementary Data (File S1).

	Genetic background concerning previously described non-synonymous SNPs associated with antimalarial drug resistance				
**Gene association**	**Gene name**	**Gene ID**	**SMT010**	**Dd2**	**References for gene association**
Association with ART resistance validated by gene editing and RSA$^{0\text{-}3h}$	*k13 (kelch 13)*	PF3D7_1343700	WT	WT	(1, 2)
	coronin	PF3D7_1251200	WT	WT	(3)
	ap2μ (μ-subunit of the AP2 vesicular trafficking complex)	PF3D7_1218300	WT	WT	(4)
	ubp1 (putative ubiquitin hydrolase 1)	PF3D7_0104300	WT	WT	
Potential association with ART resistance (in genome wide association studies)	*arps10 (apicoplast ribosomal protein S10)*	PF3D7_1460900	WT	WT	(5)
	fd (ferredoxin)	PF3D7_1318100	WT	D193Y	
	pib7(phosphoinositide-binding protein)	PF3D7_0720700	WT	WT	
	NLI interacting factor-like phosphatase (NIF4 or pph)	PF3D7_1012700	WT	V1157L	
	crt (chloroquine resistance transporter)	PF3D7_0709000	WT	N326S, I356T	
	mdr2 (multidrug resistance protein 2)	PF3D7_1447900	WT	T484I	
	atg18 (autophagy related gene 18)	PF3D7_1012900	WT	T38I	(6)
Association with resistance to other antimalarial drugs	*crt (chloroquine resistance transporter)*	PF3D7_0709000	WT	K76T	(7)
	mdr1(multidrug resistance protein 1)	PF3D7_0523000	N86Y, Y184F	WT	
	dhfr (dihydrofolate reductase)	PF3D7_0417200	WT	N51I, C59R, S108N	(8)
	dhps (dihydropteroate synthase)	PF3D7_0810800	G437A*	S436F, A613S, 437G*	(9)
	mdr2 (multidrug resistance protein 2)	PF3D7_1447900	F423Y	F423Y	(10)
	cytb (cytochrome b)	mal_mito_3	WT	WT	(11)
	carl (cyclic amine resistance locus)	PF3D7_1113300	WT	WT	(12)
	conserved Plasmodium protein, unknown function	PF3D7_0108300	E391D	E391D	
	serine/threonine protein kinase, putative (ARK2)	PF3D7_0309200	WT	H711Y	
	conserved Plasmodium protein, unknown function	PF3D7_0317300	WT	N1241D, E3168G	

Potential association with ART resistance acquisition	*phosphatidylinositol 3-kinase (PI3K)*	PF3D7_0515300	WT	Q431K, D599N	(13)
	conserved Plasmodium protein, unknown function	PF3D7_0525200	WT	L447F	
	RAP protein, putative	PF3D7_0526000	D798N	WT	
	6-cysteine protein (P12p)	PF3D7_0612800	WT	N228D	
	phosphoinositide-binding protein	PF3D7_0720700	WT	D1711N	
	conserved Plasmodium protein, unknown function	PF3D7_0723900	WT	E734K	
	chloroquine resistance transporter (CRT)	PF3D7_0709000	WT	N326S, I356T, R371I	
	conserved Plasmodium protein, unknown function	PF3D7_0808000	N634H	WT	
	conserved Plasmodium protein, unknown function	PF3D7_0809400	L660F	L660F	
	conserved Plasmodium protein, unknown function	PF3D7_0817600	WT	K142R	
	conserved Plasmodium protein, unknown function	PF3D7_0908200	WT	D1271H	
	conserved Plasmodium protein, unknown function	PF3D7_0912000	WT	R81K, P1056S, Y1490H	
	conserved Plasmodium protein, unknown function	PF3D7_0916400	WT	Q2430E	
	tetratricopeptide repeat family protein	PF3D7_0921600	WT	Q1021K	
	conserved Plasmodium protein, unknown function	PF3D7_1008100	I1677V	WT	
	NLI interacting factor-like phosphatase (NIF4)	PF3D7_1012700	WT	Y1133N, V1157L	
	autophagy-related protein 18 (ATG18)	PF3D71012900	WT	T38I	
	tRNA pseudouridine synthase	PF3D7_1018000	I227N, S229I	I227N, S229I	
	autophagy-related protein 7 (ATG7)	PF3D7_1126100	N994K	L361M, G700E	
	conserved Plasmodium protein, unknown function	PF3D7_1140700	N552D	N786Y, K2102R	
	DNA repair endonuclease (ERCC4)	PF3D7_1368800	P161S, F165L, K167N, N212K	P161S, F165L, K167N, N212K, D1628N	
	conserved Plasmodium protein, unknown function	PF3D7_1312800	F772Y	F772Y, A933E	
	ferredoxin	PF3D7_1318100	WT	D193Y	
	conserved Plasmodium protein, unknown function	PF3D7_1318300	WT	D1193E	
	conserved Plasmodium protein, unknown function	PF3D7_1322400	E193V	WT	
	alpha/beta-hydrolase	PF3D7_1328500	WT	N853K	
	nucleoside transporter 1 (NT1)	PF3D7_1347200	WT	F394L	
	AAA family ATPase	PF3D7_1412700	K330N	WT	
	conserved Plasmodium protein, unknown function	PF3D7_1423500	N238D	N190D	
	ADP-dependent DNA helicase RecQ (WRN)	PF3D7_1429900	WT	N733K	
	U3 small nucleolar RNA-associated protein 12, (UTP12)	PF3D7_1448000	WT	H1271R	

	conserved Plasmodium protein, unknown function	PF3D7_1452600	WT	D2419N	
	conserved Plasmodium protein, unknown function	PF3D7_1466200	WT	V872I, A875T	
	derlin-1 (DER1-1)	PF3D7_1468500	WT	S102C	
**Genetic background concerning genes whose inactivation or disruption has been associated with ART resistance**					
Potential association with ART resistance (role of SNPs not validated)	*fp2a (falcipain 2a)*	PF3D7_1115700	WT	K255R, N257K, N257D	(14)
	kic4 (Kelch13 interaction candidate 4)	PF3D7_1246300	WT	WT	(15)
	kic5 (kelch13 interaction candidate)	PF3D7_1138700	H138Y, E497Q, S1721A	E497Q, S633N, S1721A	
	kic7 (kelch13 interaction candidate 7)	PF3D7_0813000	WT	WT	
	mca2 (metacaspase 2)	PF3D7_1438400	M136V, V139M, V145M, M148V, V151M, M154V, V160M, M166V, D226E	N1085D, D1116H	
	ap2μ (μ-subunit of the AP2 vesicular trafficking complex)	PF3D7_1218300	WT	WT	
	eps15 (eps15-like protrein)	PF3D7_1025000	F486S, N559D, N560D, N565D, N566D	F486S, N559D, N560D, N565D, N566D	
	ubp1 (putative ubiquitin hydrolase 1)	PF3D7_0104300	Y615H, N759K, K764N, D777G, D795N, D797N, N798D, K1193T, D1525E, K1914N, E1915K, R2238K, K2809N, N2813K, G2814C, Y2822C	Y615H, D788N, N789K, E1011K, R1133S, K1914N, E1915K, R2238K, F2333L	

* In the Pf3D7 reference line, DHPS have a glycine at the position 437 (437G) but this amino acid is associated with sulfadoxine resistance (alternative amino acid: alanine). Consequently, the strain SMT010 with the mutation G437A is sulfadoxine-sensitive and the strain Dd2 is sulfadoxine-resistant.

Table S7: List of K13 protein mutations located in the BTB/POZ domain, reported in the literature and their involvement in ART resistance.

Mutation	Origin	Association with ART resistance	References
I352T	China-Myanmar border	Blood spot / no clinical data	(16)
	Myanmar	Blood spot / no clinical data	(17)
**D353Y**	Vietnam	**$PCt_{1/2}$ > 5 h**	(5, 18)
I354V	Kenya	Blood spot / no clinical data	(19)
G357V	Senegal	Blood spot / no clinical data	(20)
L368I	Senegal	Blood spot / no clinical data	(20)
I376V	China-Myanmar border	Blood spot / no clinical data	(16)
S381N	Thailand	Blood spot / no clinical data	(18)
F395Y	South East Asia	$PCt_{1/2}$ < 5 h	(5, 18)
R398M	Senegal	Blood spot / no clinical data	(20)
E401Q	Eritrea	Blood spot / no clinical data	(21)
R411K	Myanmar	Blood spot / no clinical data	(17)
I416V	Africa	Blood spot / no clinical data	(22)
	Tanzania	Blood spot / no clinical data	(23)
I416M	Africa	Blood spot / no clinical data	(22)
S423I	Senegal	Blood spot / no clinical data	(20)
L429F	Senegal	Blood spot / no clinical data	(20)
E433D	Tanzania, Senegal	Blood spot / no clinical data	(18, 23)
I437T	Thai-Myanmar border	Blood spot / no clinical data	(24)

Note – $PCt_{1/2}$: parasite clearance half-life.

Table S8: WGS statistics of *P. falciparum* DNA extracted from SMT010, SMT010p19, Dd2 and Dd2-D8^{P413A}.

**Sample**	**Total Reads**	***P. falciparum* mapped reads (%)**	**Mean coverage**	**% genome covered at 10X**
SMT010	24,712,022	24,443,458 (98.91)	148.79	97.95
SMT010p19	15,764,944	15,574,702 (98.79)	80.29	94.91
Dd2	14,791,629	14,564,576 (98.46)	72.67	95.05
Dd2-D8^{P413A}	20,591,021	20,341,891 (98.79)	102.94	96.63

Supplemental file S2:

Molecular dynamics simulations on PfK13 WT and P413A mutant structures

1. Structure preparation and molecular dynamics simulation set up

The K13 BTB/POZ-Propeller crystallographic structure was retrieved from the Protein Data Bank (PDB) repository, PDB ID: 4zgc (http://pfam.xfam.org/structure/4YY8). The P413A mutation was introduced *in silico* using the *swapaa* function of UCSF Chimera by substituting the residue with the most probable rotameric conformation (**Figure X**) (25). Importantly, we supposed here that the P413A mutant structure was similarly folded to the WT one before running simulations. All missing atoms were then added using Swiss PDB Viewer (26). Both WT and mutant structures were checked for quality using MolProbity (27), showing no outliers and > 98 % of residues (including the *in silico* introduced mutation) in favored regions.

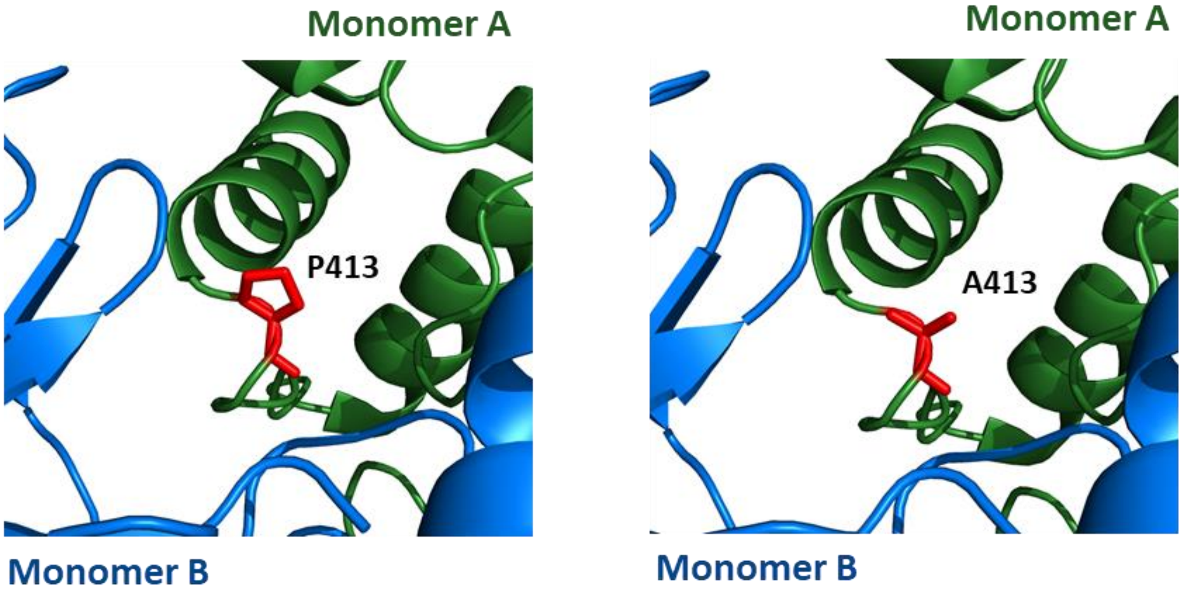

Figure X *In silico* mutation of P413 to A413 with the most probable rotameric conformation. The structure is shown as cartoon, while position 413 is shown in stick. Monomers A and B are respectively colored in green and blue, and shown as cartoon. Position 413 is colored in red and shown as stick.

The pipeline of the molecular dynamics simulations and subsequent analyses were fully described in the materials and methods section.

2. Analysis of molecular dynamics trajectories.

Since we obtained very similar observations on monomeric and homodimeric simulations (data not shown), we only present here the results observed for the monomeric state. After 100 ns of simulation, protein trajectories were very similar between WT and mutant P413A structures as shown by the Cα-based root mean square deviations (RMSDs; **Figure YA**) and the radius of gyrate (**Figure YB**), suggesting the absence of drastic conformational alterations in the mutant P413A in a context of well-folded protein. At the amino acid level, P413 and A413 were found to make no hydrogen bonds with any other PfK13 positions (**Figure YC**). Exploration of the Cα-based root mean square fluctuations (RMSFs) also revealed no evident modification in the mobility of the residues in the mutant P413A when compared to the WT (**Figure YD**), consistently with the similar contact maps between WT and mutant structures (**Figure YE**). The turn formed by P413 was maintained in the mutant P413A structure (**Figure YF**). Finally, P413A mutation did not alter the electrostatic potential at the surface of PfK13 BTB and propeller domains, which was very electronegative similarly to the WT structure (**Figure YG**).

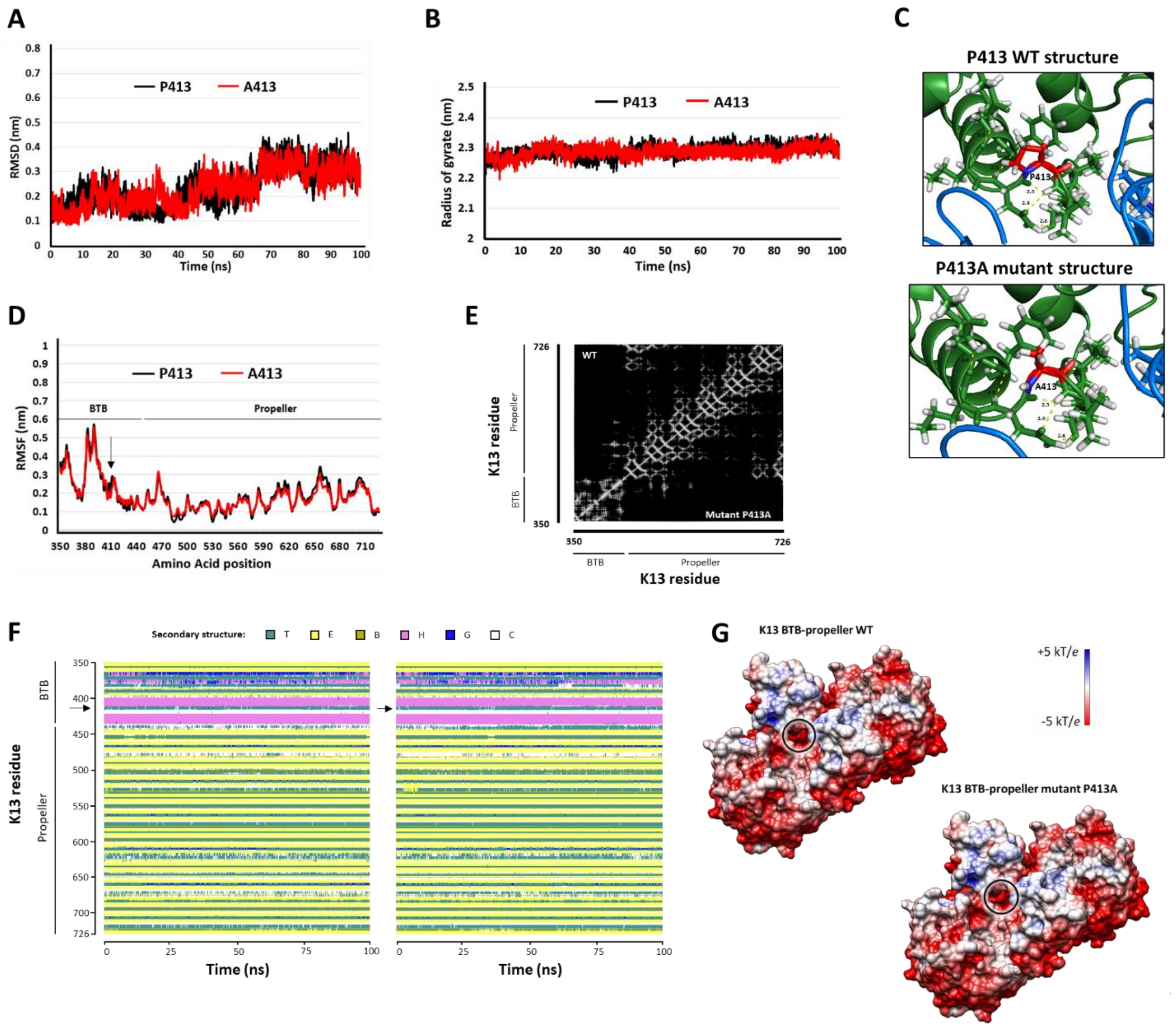

Figure Y - Structural analyses of P413A mutation on the PfK13 BTB/POZ-Propeller structure as assessed by molecular dynamics simulations. The structure is shown as cartoon, while position 413 is shown in stick. Monomers A and B are respectively colored in green and blue, and shown as cartoon. Position 413 is colored in red and shown as stick. **(A)** Cα-based root mean square deviations (RMSDs) of WT P413 and mutant A413 monomer PfK13 structure. The RMSD was measured at every 10 ps of the 100 ns molecular dynamics simulations. WT and mutant structures are indicated in black and red, respectively. (**B**) Radius of gyration (Rg) of WT P413 and mutant A413 monomer PfK13 structure. Rg is an indicator of protein structure compactness Colors are the same as in A. (**C**) Stable hydrogen bonds around residue 413. Only bonds observed in at least 50% of the simulations are indicated. No hydrogen bond was formed with P413 or A413 and other residues. (**D**) Cα-based root mean square fluctuations (RMSFs) at each residue of WT P413 and mutant A413 monomer PfK13 structure. RMSF values were calculated from the 100 ns molecular dynamics simulations. The arrow indicates the location of the residue 413. Colors are the same as in A. (**E**) Contact map between each pair of residues in WT (*top left*) and mutant A413 (*bottom right*) structures. White colour indicates a contact for a pair of residues. Only contacts observed in at least 50 % of the

simulations are indicated. (**F**) Evolution of secondary structures for each residue of WT and mutant P413A PfK13 BTB-Propeller structures during 100 ns simulations. The arrow indicates the location of position 413. T, Turn; E, Extended configuration (β-sheet); B, Isolated bridge; H, α-helix; G, 3-10 helix; C, Coil. (**G**) Electrostatic surface potential of WT P413 and mutant A413 homodimeric PfK13 BTB-Propeller structures, estimated with the APBS method. Electrostatic potential values are in units of kT/e at 298 K, on a scale of −5 kT/e (red) to +5 kT/e (blue). White color indicates a neutral potential.

3. Interpretation

On the WT structure, as a proline, position 413 clearly forms a turn maintained during the whole simulations (**Figure YF**). During the folding process of P413A mutant structure, this turn may likely be lost. However, this turn was still present in our P413A simulations even in the absence of any structural constraints (**Figure YF**). This may be explained by the *in silico* introduction of the mutation from a well-folded, WT structure that includes the turn. As a consequence, the absence of conformational changes in the P413A mutant structure in our simulations may not be a reflection of the real conformation.

Supplemental references:

1. Ariey F, Witkowski B, Amaratunga C, Beghain J, Langlois A-C, Khim N, Kim S, Duru V, Bouchier C, Ma L, Lim P, Leang R, Duong S, Sreng S, Suon S, Chuor CM, Bout DM, Ménard S, Rogers WO, Genton B, Fandeur T, Miotto O, Ringwald P, Le Bras J, Berry A, Barale J-C, Fairhurst RM, Benoit-Vical F, Mercereau-Puijalon O, Ménard D. 2014. A molecular marker of artemisinin-resistant Plasmodium falciparum malaria. Nature 505:50–55.

2. Straimer J, Gnadig NF, Witkowski B, Amaratunga C, Duru V, Ramadani AP, Dacheux M, Khim N, Zhang L, Lam S, Gregory PD, Urnov FD, Mercereau-Puijalon O, Benoit-Vical F, Fairhurst RM, Menard D, Fidock DA. 2015. K13-propeller mutations confer artemisinin resistance in Plasmodium falciparum clinical isolates. Science 347:428–431.

3. Demas AR, Sharma AI, Wong W, Early AM, Redmond S, Bopp S, Neafsey DE, Volkman SK, Hartl DL, Wirth DF. 2018. Mutations in *Plasmodium falciparum* actin-binding protein coronin confer reduced artemisinin susceptibility. Proc Natl Acad Sci USA 115:12799–12804.

4. Henrici RC, van Schalkwyk DA, Sutherland CJ. 2020. Modification of pfap2□ and pfubp1 Markedly Reduces Ring- Stage Susceptibility of Plasmodium falciparum to Artemisinin In Vitro. Antimicrobial Agents and Chemotherapy 64:9.

5. Miotto O, Amato R, Ashley EA, MacInnis B, Almagro-Garcia J, Amaratunga C, Lim P, Mead D, Oyola SO, Dhorda M, Imwong M, Woodrow C, Manske M, Stalker J, Drury E, Campino S, Amenga-Etego L, Thanh T-NN, Tran HT, Ringwald P, Bethell D, Nosten F, Phyo AP, Pukrittayakamee S, Chotivanich K, Chuor CM, Nguon C, Suon S, Sreng S, Newton PN, Mayxay M, Khanthavong M, Hongvanthong B, Htut Y, Han KT, Kyaw MP, Faiz MA, Fanello CI, Onyamboko M, Mokuolu OA, Jacob CG, Takala-Harrison S, Plowe

CV, Day NP, Dondorp AM, Spencer CCA, McVean G, Fairhurst RM, White NJ, Kwiatkowski DP. 2015. Genetic architecture of artemisinin-resistant Plasmodium falciparum. Nat Genet 47:226–234.

6. Breglio KF, Amato R, Eastman R, Lim P, Sa JM, Guha R, Ganesan S, Dorward DW, Klumpp-Thomas C, McKnight C, Fairhurst RM, Roberts D, Thomas C, Simon AK. 2018. A single nucleotide polymorphism in the Plasmodium falciparum atg18 gene associates with artemisinin resistance and confers enhanced parasite survival under nutrient deprivation. Malar J 17:391.

7. Djimdé A, Kayentao K, Su X-Z, Wellems TE. 2001. A Molecular Marker for Chloroquine-Resistant Falciparum Malaria. The New England Journal of Medicine 7.

8. Peterson DS, Walliker D, Wellems TE. 1988. Evidence that a point mutation in dihydrofolate reductase-thymidylate synthase confers resistance to pyrimethamine in falciparum malaria. Proceedings of the National Academy of Sciences 85:9114–9118.

9. Triglia T, Wang P, Sims PFG, Hyde JE, Cowman AF. 1998. Allelic exchange at the endogenous genomic locus in Plasmodium falciparum proves the role of dihydropteroate synthase in sulfadoxine-resistant malaria. EMBO J 17:3807–3815.

10. Briolant S, Bogreau H, Gil M, Bouchiba H, Baret E, Amalvict R, Rogier C, Pradines B. 2012. The F423Y Mutation in the *pfmdr2* Gene and Mutations N51I, C59R, and S108N in the *pfdhfr* Gene Are Independently Associated with Pyrimethamine Resistance in Plasmodium falciparum Isolates. Antimicrob Agents Chemother 56:2750–2752.

11. Korsinczky M, Chen N, Kotecka B, Saul A, Rieckmann K, Cheng Q. 2000. Mutations in Plasmodium falciparumCytochrome b That Are Associated with Atovaquone Resistance

Are Located at a Putative Drug-Binding Site. Antimicrob Agents Chemother 44:2100–2108.

12. LaMonte G, Lim MY-X, Wree M, Reimer C, Nachon M, Corey V, Gedeck P, Plouffe D, Du A, Figueroa N, Yeung B, Bifani P, Winzeler EA. 2016. Mutations in the *Plasmodium falciparum* Cyclic Amine Resistance Locus (PfCARL) Confer Multidrug Resistance. mBio 7:e00696-16, /mbio/7/4/e00696-16.atom.

13. Dwivedi A, Reynes C, Kuehn A, Roche DB, Khim N, Hebrard M, Milanesi S, Rivals E, Frutos R, Menard D, Mamoun CB, Colinge J, Cornillot E. 2017. Functional analysis of Plasmodium falciparum subpopulations associated with artemisinin resistance in Cambodia. Malar J 16:493.

14. Siddiqui FA, Cabrera M, Wang M, Brashear A, Kemirembe K, Wang Z, Miao J, Chookajorn T, Yang Z, Cao Y, Dong G, Rosenthal PJ, Cui L. 2018. Plasmodium falciparum Falcipain-2a Polymorphisms in Southeast Asia and Their Association With Artemisinin Resistance. The Journal of Infectious Diseases 218:434–442.

15. Birnbaum J, Scharf S, Schmidt S, Jonscher E, Hoeijmakers WAM, Flemming S, Toenhake CG, Schmitt M, Sabitzki R, Bergmann B, Fröhlke U, Mesén-Ramírez P, Blancke Soares A, Herrmann H, Bártfai R, Spielmann T. 2020. A Kelch13-defined endocytosis pathway mediates artemisinin resistance in malaria parasites. Science 367:51–59.

16. Wang Z, Shrestha S, Li X, Miao J, Yuan L, Cabrera M, Grube C, Yang Z, Cui L. 2015. Prevalence of K13-propeller polymorphisms in Plasmodium falciparum from China-Myanmar border in 2007–2012. Malar J 14:168.

17. Win AA, Imwong M, Kyaw MP, Woodrow CJ, Chotivanich K, Hanboonkunupakarn B, Pukrittayakamee S. 2016. K13 mutations and pfmdr1 copy number variation in Plasmodium falciparum malaria in Myanmar. Malar J 15:110.

18. MalariaGEN, Ahouidi A, Ali M, Almagro-Garcia J, Amambua-Ngwa A, Amaratunga C, Amato R, Amenga-Etego L, Andagalu B, Anderson TJC, Andrianaranjaka V, Apinjoh T, Ariani C, Ashley EA, Auburn S, Awandare G, Ba H, Baraka V, Barry AE, Bejon P, Bertin GI, Boni MF, Borrmann S, Bousema T, Branch O, Bull PC, Busby GBJ, Chookajorn T, Chotivanich K, Claessens A, Conway D, Craig A, D'Alessandro U, Dama S, Day NP, Denis B, Diakite M, Djimdé A, Dolecek C, Dondorp AM, Drakeley C, Drury E, Duffy P, Echeverry DF, Egwang TG, Erko B, Fairhurst RM, Faiz A, Fanello CA, Fukuda MM, Gamboa D, Ghansah A, Golassa L, Goncalves S, Hamilton WL, Harrison GLA, Hart L, Henrichs C, Hien TT, Hill CA, Hodgson A, Hubbart C, Imwong M, Ishengoma DS, Jackson SA, Jacob CG, Jeffery B, Jeffreys AE, Johnson KJ, Jyothi D, Kamaliddin C, Kamau E, Kekre M, Kluczynski K, Kochakarn T, Konaté A, Kwiatkowski DP, Kyaw MP, Lim P, Lon C, Loua KM, Maïga-Ascofaré O, Malangone C, Manske M, Marfurt J, Marsh K, Mayxay M, Miles A, Miotto O, Mobegi V, Mokuolu OA, Montgomery J, Mueller I, Newton PN, Nguyen T, Nguyen T-N, Noedl H, Nosten F, Noviyanti R, Nzila A, Ochola-Oyier LI, Ocholla H, Oduro A, Omedo I, Onyamboko MA, Ouedraogo J-B, Oyebola K, Pearson RD, Peshu N, Phyo AP, Plowe CV, Price RN, Pukrittayakamee S, Randrianarivelojosia M, Rayner JC, Ringwald P, Rockett KA, Rowlands K, Ruiz L, Saunders D, Shayo A, Siba P, Simpson VJ, Stalker J, Su X, Sutherland C, Takala-Harrison S, Tavul L, Thathy V, Tshefu A, Verra F, Vinetz J, Wellems TE, Wendler J, White NJ, Wright I, Yavo W, Ye H. 2021. An open dataset of Plasmodium falciparum genome variation in 7,000 worldwide samples. Wellcome Open Res 6:42.

19. Wamae K, Okanda D, Ndwiga L, Osoti V, Kimenyi KM, Abdi AI, Bejon P, Sutherland C, Ochola-Oyier LI. 2019. No Evidence of *Plasmodium falciparum k13* Artemisinin Resistance-Conferring Mutations over a 24-Year Analysis in Coastal Kenya but a Near Complete Reversion to Chloroquine-Sensitive Parasites. Antimicrob Agents Chemother 63:e01067-19, /aac/63/12/AAC.01067-19.atom.

20. Talundzic E, Ndiaye YD, Deme AB, Olsen C, Patel DS, Biliya S, Daniels R, Vannberg FO, Volkman SK, Udhayakumar V, Ndiaye D. 2017. Molecular Epidemiology of Plasmodium falciparum kelch13 Mutations in Senegal Determined by Using Targeted Amplicon Deep Sequencing. Antimicrob Agents Chemother 61:e02116-16, e02116-16.

21. L'Episcopia M, Kelley J, Patel D, Schmedes S, Ravishankar S, Menegon M, Perrotti E, Nurahmed AM, Talha AA, Nour BY, Lucchi N, Severini C, Talundzic E. 2020. Targeted deep amplicon sequencing of kelch 13 and cytochrome b in Plasmodium falciparum isolates from an endemic African country using the Malaria Resistance Surveillance (MaRS) protocol. Parasites Vectors 13:137.

22. MalariaGEN. 2016. Genomic epidemiology of artemisinin resistant malaria. eLife 5:e08714.

23. Ishengoma DS, Mandara CI, Francis F, Talundzic E, Lucchi NW, Ngasala B, Kabanywanyi AM, Mahende MK, Kamugisha E, Kavishe RA, Muro F, Mohamed A, Mandike R, Mkude S, Chacky F, Paxton L, Greer G, Kitojo CA, Njau R, Martin T, Venkatesan M, Warsame M, Halsey ES, Udhayakumar V. 2019. Efficacy and safety of artemether-lumefantrine for the treatment of uncomplicated malaria and prevalence of Pfk13 and Pfmdr1 polymorphisms after a decade of using artemisinin-based combination therapy in mainland Tanzania. Malar J 18:88.

24. Phompradit P, Chaijaroenkul W, Muhamad P, Na-Bangchang K. 2019. K13 propeller domain mutations and pfmdr1 amplification in isolates of Plasmodium falciparum collected from Thai-Myanmar border area in 2006-2010. FOLIA PARASIT 66.

25. Pettersen EF, Goddard TD, Huang CC, Couch GS, Greenblatt DM, Meng EC, Ferrin TE. 2004. UCSF Chimera?A visualization system for exploratory research and analysis. J Comput Chem 25:1605–1612.

26. Guex N, Peitsch MC. 1997. SWISS-MODEL and the Swiss-PdbViewer: an environment for comparative protein modeling. Electrophoresis 18:2714–2723.

27. Chen VB, Arendall WB, Headd JJ, Keedy DA, Immormino RM, Kapral GJ, Murray LW, Richardson JS, Richardson DC. 2010. MolProbity: all-atom structure validation for macromolecular crystallography. Acta Crystallogr D Biol Crystallogr 66:12–21.
